# A replica exchange Monte Carlo algorithm for protein folding in the HP model

**DOI:** 10.1186/1471-2105-8-342

**Published:** 2007-09-17

**Authors:** Chris Thachuk, Alena Shmygelska, Holger H Hoos

**Affiliations:** 1 School of Computing Science, Simon Fraser University, Burnaby, B.C., V5A 1S6, Canada; 2 Department of Structural Biology, Stanford University, Stanford, CA, 94305, USA; 3 Department of Computer Science, University of British Columbia, B.C., V6T 1Z4, Canada

## Abstract

**Background:**

The *ab initio *protein folding problem consists of predicting protein tertiary structure from a given amino acid sequence by minimizing an energy function; it is one of the most important and challenging problems in biochemistry, molecular biology and biophysics. The *ab initio *protein folding problem is computationally challenging and has been shown to be NP
 MathType@MTEF@5@5@+=feaafiart1ev1aaatCvAUfKttLearuWrP9MDH5MBPbIqV92AaeXatLxBI9gBaebbnrfifHhDYfgasaacH8akY=wiFfYdH8Gipec8Eeeu0xXdbba9frFj0=OqFfea0dXdd9vqai=hGuQ8kuc9pgc9s8qqaq=dirpe0xb9q8qiLsFr0=vr0=vr0dc8meaabaqaciaacaGaaeqabaqabeGadaaakeaat0uy0HwzTfgDPnwy1egaryqtHrhAL1wy0L2yHvdaiqaacqWFneVtcqqGqbauaaa@3961@-hard even when conformations are restricted to a lattice. In this work, we implement and evaluate the replica exchange Monte Carlo (REMC) method, which has already been applied very successfully to more complex protein models and other optimization problems with complex energy landscapes, in combination with the highly effective *pull move *neighbourhood in two widely studied Hydrophobic Polar (HP) lattice models.

**Results:**

We demonstrate that REMC is highly effective for solving instances of the square (2D) and cubic (3D) HP protein folding problem. When using the pull move neighbourhood, REMC outperforms current state-of-the-art algorithms for most benchmark instances. Additionally, we show that this new algorithm provides a larger ensemble of ground-state structures than the existing state-of-the-art methods. Furthermore, it scales well with sequence length, and it finds significantly better conformations on long biological sequences and sequences with a provably unique ground-state structure, which is believed to be a characteristic of real proteins. We also present evidence that our REMC algorithm can fold sequences which exhibit significant interaction between termini in the hydrophobic core relatively easily.

**Conclusion:**

We demonstrate that REMC utilizing the pull move neighbourhood significantly outperforms current state-of-the-art methods for protein structure prediction in the HP model on 2D and 3D lattices. This is particularly noteworthy, since so far, the state-of-the-art methods for 2D and 3D HP protein folding – in particular, the pruned-enriched Rosenbluth method (PERM) and, to some extent, Ant Colony Optimisation (ACO) – were based on chain growth mechanisms. To the best of our knowledge, this is the first application of REMC to HP protein folding on the cubic lattice, and the first extension of the pull move neighbourhood to a 3D lattice.

## Background

The *ab initio *protein folding problem concerns the prediction of the three dimensional functional state, *i.e.*, the *native *fold, of a protein given only its sequence information. A successful method for solving this problem would have far reaching implications in many fields including structural biology, genetics and medicine. Current laboratory techniques for protein structure determination are both costly and time consuming. In the current era of high throughput sequencing, it is infeasible to rely exclusively on time and labor-intensive experimental structure determination techniques, such as X-ray crystallography and nuclear magnetic resonance, for characterizing the protein products of newly discovered genes; there is a clear need for effective and efficient computational protein structure prediction programs. However, even for simplified protein models that use lattices to discretize the conformational space, the *ab initio *protein structure prediction problem has been shown to be NP
 MathType@MTEF@5@5@+=feaafiart1ev1aaatCvAUfKttLearuWrP9MDH5MBPbIqV92AaeXatLxBI9gBaebbnrfifHhDYfgasaacH8akY=wiFfYdH8Gipec8Eeeu0xXdbba9frFj0=OqFfea0dXdd9vqai=hGuQ8kuc9pgc9s8qqaq=dirpe0xb9q8qiLsFr0=vr0=vr0dc8meaabaqaciaacaGaaeqabaqabeGadaaakeaat0uy0HwzTfgDPnwy1egaryqtHrhAL1wy0L2yHvdaiqaacqWFneVtcqqGqbauaaa@3961@-hard [1-3], and a polynomial-time algorithm is therefore unlikely to exist.

One of the most prevalently studied abstractions of the *ab initio *protein structure prediction problem is Dill's *hydrophobic polar *(HP) model. Many algorithms have been formulated to address the protein folding problem using two dimensional (2D) and three dimensional (3D) HP models on a variety of lattices (see, *e.g.*, [4-12]). In this study, we restrict our attention to those HP models that embed all protein folds into the 2D square lattice or the 3D cubic lattice. Many of these algorithms can be classified primarily as *construction based *(or *chain growth*) algorithms, which determine folds by sequentially placing residues onto the lattice. Among these, the pruned enriched Rosenbluth method (PERM) [13] has been particularly successful in finding optimal conformations for standard benchmark sequences in both 2D and 3D. PERM is a Monte Carlo based chain growth algorithm that iteratively constructs partial conformations; it is heavily based on mechanisms for pruning unfavourable folds and for enriching promising partial conformations, to facilitate their further exploration.

Despite being one of the most successful algorithms for *ab initio *protein structure prediction in the 2D and 3D HP models, PERM – like all other currently known algorithms for this problem – is not dominant in every instance. In the work of Shmygelska and Hoos [9] it was shown that PERM has great difficulty folding proteins which have a hydrophobic core located in the middle and not at one of the ends of the sequence, as is the case when the core is formed from interacting termini. We note that an earlier version of PERM [14], capable of initiating search at non-terminus positions, was previously proposed and may be more effective in folding these types of sequences. However, to the best of our knowledge, no comparison has been made with the most recent version of PERM or other protein folding algorithms.

Shmygelska and Hoos proposed an ant colony optimization algorithm, ACO-HPPFP-3, which employs both construction and local search phases on complete conformations [9]. Ant Colony Optimisation (ACO) is a population-based stochastic search method for solving a wide range of combinatorial optimisation problems. ACO is based on the concept of stigmergy – indirect communication between members of a population through interaction with the environment. From the computational point of view, ACO is an iterative construction search method in which a population of simple agents ('ants') repeatedly constructs candidate solutions to a given problem instance; this construction process is probabilistically guided by heuristic information on the problem instance as well as by a shared memory containing experience gathered by the ants in previous iterations of the search process ('pheromone trails') [15]. The ACO-HPPFP-3 algorithm combines a relatively straight-forward application of the general ACO method to the 2D and 3D HP protein structure prediction problem with specific local search procedures that are used to optimize the conformations constructed by the ants.

In the 2D case, ACO-HPPFP-3 was shown to be competitive with PERM on many benchmark instances and dominant on proteins whose hydrophobic core is located in the middle of the sequence. Other attempts at the problem use local search methods on complete conformations, including the GTabu algorithm [7]. This method utilizes the generic tabu search algorithm from the Human Guided Search (HuGS) framework [16]. GTabu was shown to find conformations with the lowest known energy for several benchmark instances in the 2D case. This was primarily made possible by using a newly introduced neighbourhood consisting of so-called *pull moves*, which is also utilized in our work.

In addition to PERM, many other Monte Carlo algorithms have been devised to address the problem of *ab initio *protein structure prediction using lattice models [17-20]. A class of Monte Carlo methods known as *generalized ensemble algorithms *have been shown to be particularly effective for more complex lattices and for the off-lattice case [5,21-24]. Classical Monte Carlo search methods for protein structure prediction typically sample conformations according to the Boltzmann distribution in energy space. In generalized ensemble algorithms, random walks in other dimensions, such as temperature, can also be realized. This is the case for replica exchange Monte Carlo (REMC) algorithms, which maintain many independent replicas of potential solutions, *i.e.*, protein conformations. Each replica is set at a different temperature and locally runs a Markov process sampling from the Boltzmann distribution in energy space. A random walk in temperature space is achieved by periodic exchanges of conformations at neighbouring temperatures. REMC appears to have been discovered independently by various researchers [25-28] and is also known as parallel tempering, multiple Markov chain Monte Carlo and exchange Monte Carlo search. REMC has been shown to be highly effective in high dimensional search problems with rugged landscapes containing many local minima. Initially this was demonstrated in an application to spin glass systems [26,29]. REMC has also been applied to the off-lattice protein folding problem [21-24,30-34]. Furthermore, it was previously used for folding proteins on the 2D square lattice in a study by Irbäck [23] and to the face-centred cubic lattice in the work of Gront *et al *[5]. However, to the best of our knowledge, no extensive study of the REMC algorithm in the HP model on the cubic lattice has been undertaken. The remainder of this paper is structured as follows. First, we formally introduce the *hydrophobic polar *model and describe in detail the two search neighbourhoods (move sets) utilized later in this work. Next, we present the general REMC method followed by the three instantiations we have developed for the 2D and 3D HP protein folding problem. Then, we report results from a comparative empirical performance analysis of our new algorithms *vs *PERM and ACO-HPPFP-3. The respective computational experiments are run on standard benchmark instances as well as on two new sequence sets, which we introduced to evaluate the performance of REMC when folding long sequences and sequences which have a provably unique optimal structure. We also report results from experiments involving proteins with termini interacting to form a hydrophobic core. Next, we compare the performance of our new REMC algorithms with that of GTabu. A discussion follows, in which we report empirical results regarding the effects of various parameters on the performance of our new algorithms. We close with a high-level summary of our major findings and a brief discussion of potential future work.

### The hydrophobic polar model

**Figure 1:**
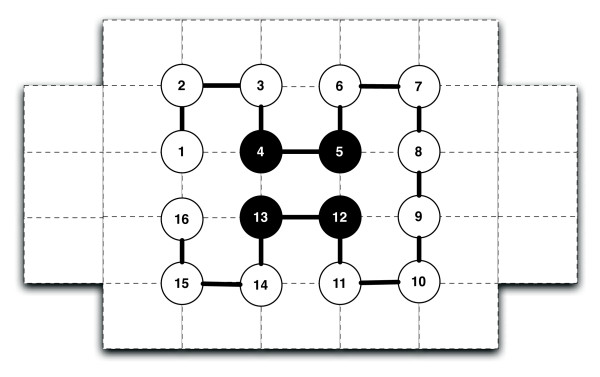
**A *ground-state conformation *in the 2D HP model**. The grid points and lines represent the 2D square lattice this conformation is embedded upon. Filled, black circles represent hydrophobic residues while unfilled circles represent polar residues. The conformation above yields an optimal energy score in the HP model of -2. The two hydrophobic contacts contributing to the score are between residues 4 and 13 and between residues 5 and 12.

The hydrophobic polar (HP) model was first introduced by Dill in 1985 [35]. In this model, amino acids are classified as either H (*hydrophobic*) or P (*polar*). Informally, a sequence of H's and P's is embedded into a lattice structure. A valid conformation of the sequence corresponds to a self-avoiding walk on the lattice. Borrowing the terminology used by Lau and Dill [36], we define *connected neighbours *as any two residues *k *and *k *+ 1 that are adjacent along the given sequence, and *topological neighbours *as residues adjacent in topological space (forming a contact) that are not also connected neighbours. The energy of a conformation can be calculated as the number of H-H contacts between topological neighbours. This is illustrated in Figure 1, which shows a conformation with energy -2 (every H-H contact contributes -1 to the total energy, while all other contacts do not contribute).

Formally, for a sequence *s *∈ Σ^*n *^with Σ = {H, P} and *n *= |*s*|, we define a conformation *c*_*i *_∈ *C*_*s *_to have energy *E*(*c*_*i*_), where *C*_*s *_is the set of all valid self-avoiding walks on some lattice *L *for sequence *s*, and *E*(*c*_*i*_) is given by the following equation:

E(ci)=∑j=1n−1∑k=j+1nNjk, withNjk={−1if j and k are both H residuesand topological neighbours;0otherwise.
 MathType@MTEF@5@5@+=feaafiart1ev1aaatCvAUfKttLearuWrP9MDH5MBPbIqV92AaeXatLxBI9gBaebbnrfifHhDYfgasaacH8akY=wiFfYdH8Gipec8Eeeu0xXdbba9frFj0=OqFfea0dXdd9vqai=hGuQ8kuc9pgc9s8qqaq=dirpe0xb9q8qiLsFr0=vr0=vr0dc8meaabaqaciaacaGaaeqabaqabeGadaaakeaafaqadeGabaaabaGaemyrauKaeiikaGIaem4yam2aaSbaaSqaaiabdMgaPbqabaGccqGGPaqkcqGH9aqpdaaeWbqaamaaqahabaGaemOta40aaSbaaSqaaiabdQgaQjabdUgaRbqabaGccqGGSaalcqqGGaaicqqG3bWDcqqGPbqAcqqG0baDcqqGObaAaSqaaiabdUgaRjabg2da9iabdQgaQjabgUcaRiabigdaXaqaaiabd6gaUbqdcqGHris5aaWcbaGaemOAaOMaeyypa0JaeGymaedabaGaemOBa4MaeyOeI0IaeGymaedaniabggHiLdaakeaacqWGobGtdaWgaaWcbaGaemOAaOMaem4AaSgabeaakiabg2da9maaceqabaqbaeaabmGaaaqaaiabgkHiTiabigdaXaqaaiabbMgaPjabbAgaMjabbccaGiabdQgaQjabbccaGiabbggaHjabb6gaUjabbsgaKjabbccaGiabdUgaRjabbccaGiabbggaHjabbkhaYjabbwgaLjabbccaGiabbkgaIjabb+gaVjabbsha0jabbIgaOjabbccaGiabbIeaijabbccaGiabbkhaYjabbwgaLjabbohaZjabbMgaPjabbsgaKjabbwha1jabbwgaLjabbohaZbqaaaqaaiabbggaHjabb6gaUjabbsgaKjabbccaGiabbsha0jabb+gaVjabbchaWjabb+gaVjabbYgaSjabb+gaVjabbEgaNjabbMgaPjabbogaJjabbggaHjabbYgaSjabbccaGiabb6gaUjabbwgaLjabbMgaPjabbEgaNjabbIgaOjabbkgaIjabb+gaVjabbwha1jabbkhaYjabbohaZjabcUda7aqaaiabicdaWaqaaiabb+gaVjabbsha0jabbIgaOjabbwgaLjabbkhaYjabbEha3jabbMgaPjabbohaZjabbwgaLjabc6caUaaaaiaawUhaaaaaaaa@AEE6@

In this model, we search for a conformation c* that minimizes the objective energy function *E*(*c*_*i*_). Such a conformation is considered a solution and is also called a *ground-state conformation *of the given protein sequence. However, many instances of the HP protein folding problem exhibit solution degeneracy, *i.e.*, have more than one minimum-energy conformation. In this sense, our definition of ground-state conformation does not imply a unique solution, but simply one that satisfies the following equation:

*E*(c*) = min{*E*(*c*_*i*_) | *c*_*i *_∈ *C*_*s*_}

Although ground-state structures in this model typically do not closely resemble the known native conformations of the respective proteins, a close correspondence has been observed in some cases [37]; this is particularly true for higher resolution lattices such as the face-centred cubic lattice.

Generally, simplified models, such as the ones considered here, are widely considered to be useful in studying certain aspects of protein folding and structure prediction, including the formation of conformations exhibiting a hydrophobic core [38,39].

### Search neighbourhoods

Local search methods (including REMC and simple Monte Carlo search) are based on the idea of iteratively improving a given candidate solution by exploring its local neighbourhood. In the protein folding problem as it is presented here, the neighbourhood of a conformation can be thought to consist of slight perturbations of the respective structure. The neighbourhoods (*move sets*) used in solving this problem specify a perturbation as a feasible change from a current conformation *c *at time *t *to a valid conformation at time *t *+ 1. Thus, the neighbourhood of a conformation *c *is a set of valid conformations *c' *that are obtained by applying a specific set of perturbations to *c*. In this study we consider two such neighbourhoods, the so-called *VSHD moves *and *pull move neighbourhoods*, for both, the 2D and 3D HP models.

#### VSHD moves

*VSHD moves*, as we will refer to them in this study, appeared early on in the simulation of polymer chains by Verdier and Stockmayer [40]. In this early work, only single residue moves were used, and the *single residue end *and *corner moves *were introduced. That work was later critiqued in a study by Hilhorst and Deutch [18], which also introduced the *two residue crankshaft move*. Gurler *et al*. combined all three types of moves into one search neighbourhood [41], which we call the *VSHD neighbourhood*.

##### End moves

**Figure 2:**
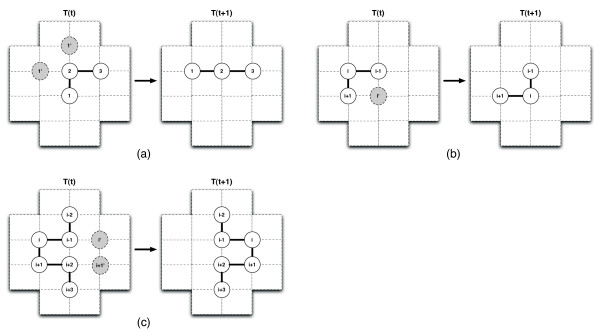
**VSHD Moves**. Residue positions are shown before the move and immediately after a successful move. *T*(*t*) denotes the state of the conformation at time *t*. In 2a there are two possible positions that residue one could be moved to, denoted by 1' in grey circles. Each position is checked in random order for availability. If a position is found to be free, the residue is moved. In 3D the same logic is followed except there is a possibility of two additional potential positions (four in total). End moves are applied on the last residue *n *in the same manner. 2b shows there to be only one potential new position for a corner move. This is also the case in 3D where the position must lie on the plane formed by *i *- 1, *i*, and *i *+ 1. 2c shows the case for a crankshaft move. In 3D, the crankshaft could potentially rotate 90° or -90°.

For a chain of length *n*, an end move can be performed on residue 1 or residue *n*. The residue is pivoted relative to its connected neighbour to a free position adjacent to that neighbour. This mechanism ensures that the chain remains connected. If more than one valid position is free, one is chosen uniformly at random. For instance, in Figure 2a, residue 1 could be moved to two possible positions on the lattice. Generally, for the 2D and 3D HP model, there are up to 2 and 4 possible moves for each of the two end residues, respectively.

##### Corner moves

A corner move can potentially be performed on any residue excluding the end residues. For a corner move to be possible, the two connected neighbours of some residue *i *must be mutually adjacent to another, unoccupied position on the lattice. Note that for both, the 2D square and the 3D cubic lattices, any two residues *i *- 1 and *i *+ 1 can share at most one adjacent lattice position. When this situation occurs, a corner is formed by residues *i *- 1, *i *and *i *+ 1. If the mutually adjacent position is empty, residue *i *can be moved to it. This is illustrated in Figure 2b for the 2D case. Overall, in 2D as well as in 3D, there are at most *n *- 2 possible corner moves for any conformation of a *n*-residue chain.

##### Crankshaft moves

A crankshaft move can occur if some residue *i *is part of a u-shaped bend in the chain, as shown in Figure 2c. Referring to this figure, the crankshaft move can be performed in 2D if positions *i' *and *i *+ 1' are empty. Crankshaft moves in 2D always involve a 180° rotation of a u-shaped structure consisting of four connected neighbours on the chain. The 3D case is handled analogously, except that the motif is rotated by either 90° or -90°, provided the appropriate positions are empty. (If both rotations are feasible, one of them is chosen uniformly at random). Note that in the figure, the same crankshaft move can be initiated from residue *i *and *i *+ 1.

#### Pull moves

*Pull moves *have been introduced relatively recently by Lesh *et al*. [7], who used them in the context of a generic tabu search algorithm for the 2D HP protein folding problem. In the following, we will briefly introduce the central idea behind this type of move. For a formal treatment of the pull move neighbourhood and the proof of its completeness (*i.e.*, the fact that any two valid sequence conformations on the 2D square lattice can be transformed into each other by a sequence of pull moves), the reader is directed to the original paper by Lesh *et al*. [7].

**Figure 3:**
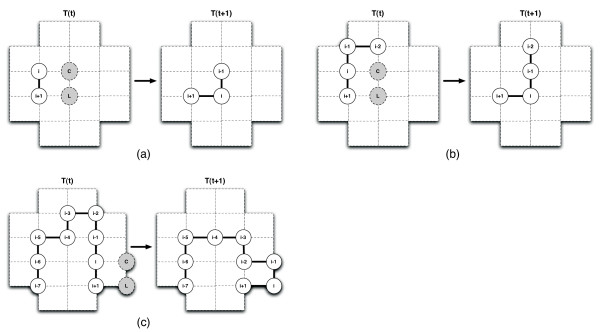
**Pull Moves**. This figure has been reproduced from [7] to illustrate the central idea behind this neighbourhood. In 3a, the simplest case where position *C *is occupied by residue *i *- 1 is shown. This move is equivalent to a corner move in the VSHD moveset. In 3b, residue *i *is moved to *L *and *i *- 1 to *C*. The chain is in a valid conformation and the move is finished. In 3c, residues *i *down to *i *- 3 must be pulled until a valid conformation is found.

Suppose at time *t *for some residue *i *there is an empty lattice position labeled *L *which is adjacent to residue *i *+ 1 and diagonally adjacent to *i*. Further consider a position mutually adjacent to *L *and *i*, labeled *C*. Using this labeling, a square is formed by residues *i*, *i *+ 1, *L *and *C*, as illustrated in Figure 3a. A pull move can only proceed if *C *is either empty or occupied by residue *i *- 1.

The simplest case occurs when *C *is occupied by residue *i *- 1, in which case the entire move consists of moving residue *i *to location *L*. Note that this move, which is illustrated in Figure 3a, is equivalent to the previously introduced corner move. When *C *is not occupied by residue *i *- 1, *i *is moved to *L *and *i *- 1 is moved to *C*. If residue *i *- 2 is adjacent to position *C*, this second operation completes the pull move. This case is illustrated in Figure 3b.

If, however, residue *i *- 2 is adjacent to position *C*, the chain is still not in a valid conformation at this point, and in this case, the following procedure is used. Using the notation by Lesh *et al*. [7], starting with residue *j *= *i *- 2, let (*x*_*j*_(*t *+ 1), *y*_*j*_(*t *+ 1)) = (*x*_*j*+2_(*t*), *y*_*j*+2_(*t*)) until a valid conformation has been found or residue 1 has been moved. Informally speaking, residues are successively pulled into positions that have just been vacated (as a consequence of pulling another residue) until a valid conformation has been obtained or one end of the chain is reached. Figure 3c illustrates this situation where residues *i *to *i *- 3 were pulled successively, until the valid conformation shown on the right was obtained. Note that pull moves have been described as pulling from residue *i *down to residue 1, if needed. Pulling in the opposite direction is equivalent and also valid.

When they introduced pull moves, Lesh *et al. *claimed that the resulting neighbourhood could be generalized to the 3D case. However, to the best of our knowledge no algorithm implementing pull moves for the 3D case has been published. For the 2D case, valid choices of *L *and *C *are restricted to a single plane. The generalization to 3D can consider choices of *L *and *C *in any plane containing both *i *and *i *+ 1; in the case of the 3D cubic lattice, there are two such planes. In our study presented here, we have implemented this generalization of pull moves in the context of a standard REMC algorithm, which will be described in the following.

### Replica exchange Monte Carlo search

In the following, we provide a brief introduction to replica exchange Monte Carlo search. For an in-depth description of the algorithm including its historical aspects, the reader is referred to the review of extended ensemble Monte Carlo algorithms by Iba [42], which also provides details related to simulated tempering [43] and replica Monte Carlo search [44].

Replica exchange Monte Carlo (REMC) search maintains *χ *independent replicas of a potential solution. Each of the *χ *replicas has an associated temperature value (*T*_1_, *T*_2_,..., *T*_*χ*_). Each temperature value is unique and the replicas are numbered such that *T*_1 _<*T*_2 _< ... <*T*_*χ*_. In our description of the algorithm, we will label the *χ *conformations maintained by the algorithm at any given time with the replica numbers (1 ,... *χ*,) and always associate temperature *T*_*j *_with replica *j *(for all *j *such that 1 ≤ *j *≤ *χ*). Thus, the exchange of replicas is equivalent to (and is commonly implemented as) the swap of replica labels.

Each of the *χ *replicas independently performs a simple Monte Carlo search at the respective temperature setting. The transition probability from some current conformation *c *to an alternative conformation *c' *is determined using the so-called Metropolis criterion such that

Pr[c→c′]:={1if ΔE≤0,e−ΔETotherwise.
 MathType@MTEF@5@5@+=feaafiart1ev1aaatCvAUfKttLearuWrP9MDH5MBPbIqV92AaeXatLxBI9gBaebbnrfifHhDYfgasaacH8akY=wiFfYdH8Gipec8Eeeu0xXdbba9frFj0=OqFfea0dXdd9vqai=hGuQ8kuc9pgc9s8qqaq=dirpe0xb9q8qiLsFr0=vr0=vr0dc8meaabaqaciaacaGaaeqabaqabeGadaaakeaaieGacqWFqbaucqWFYbGCcqGGBbWwcqWGJbWycqGHsgIRcuWGJbWygaqbaiabc2faDjabcQda6iabg2da9maaceqabaqbaeqabiGaaaqaaiabigdaXaqaaiabbMgaPjabbAgaMjabbccaGiabfs5aejabdweafjabgsMiJkabicdaWiabcYcaSaqaaiabdwgaLnaaCaaaleqabaWaaSaaaeaacqGHsislcqqHuoarcqWGfbqraeaacqWGubavaaaaaaGcbaGaee4Ba8MaeeiDaqNaeeiAaGMaeeyzauMaeeOCaiNaee4DaCNaeeyAaKMaee4CamNaeeyzauMaeiOla4caaaGaay5Eaaaaaa@5770@

where Δ*E *: = *E*(*c'*) - *E*(*c*) is the difference in energy between conformations *c' *and *c*, and *T *denotes the temperature of the replica.

We can represent the current state of the extended ensemble of all *χ *replicas as a vector **c **: = (*c*_1_,..., *c*_*χ*_) shown below, where *c*_*j *_is the conformation of replica *j*, which (as previously stated) runs at temperature *T*_*j*_. During replica exchange, temperature values of neighbouring replicas are swapped with a probability proportional to their energy and temperature differences. An exchange of temperatures, and therefore a relabeling of replicas, affects the state of the extended ensemble **c**. Therefore, we define an exchange between two replicas *i *and *j *more generally as a transition of the current ensemble state **c **to an altered state **c'**. We define *l*(*c*_*i*_) = *i*, the current label or replica number, for all *c*_*i*_. The probability of a transition from ensemble state **c **to state **c' **by exchanging replicas *i *and *j *is defined as:

Pr[c→c′]:=Pr[l(ci)↔l(cj)]:={1Δ≤0e−Δotherwise.
 MathType@MTEF@5@5@+=feaafiart1ev1aaatCvAUfKttLearuWrP9MDH5MBPbIqV92AaeXatLxBI9gBaebbnrfifHhDYfgasaacH8akY=wiFfYdH8Gipec8Eeeu0xXdbba9frFj0=OqFfea0dXdd9vqai=hGuQ8kuc9pgc9s8qqaq=dirpe0xb9q8qiLsFr0=vr0=vr0dc8meaabaqaciaacaGaaeqabaqabeGadaaakeaafaqabeGadaaabaacbiGae8huaaLae8NCaiNaei4waSfcbmGae43yamMaeyOKH4Qaf43yamMbauaacqGGDbqxaeaacqGG6aGocqGH9aqpaeaacqWFqbaucqWFYbGCcqGGBbWwcqWGSbaBcqGGOaakcqWGJbWydaWgaaWcbaGaemyAaKgabeaakiabcMcaPiabgsziRkabdYgaSjabcIcaOiabdogaJnaaBaaaleaacqWGQbGAaeqaaOGaeiykaKIaeiyxa0fabaaabaGaeiOoaOJaeyypa0dabaWaaiqabeaafaqaaeGacaaabaGaeGymaedabaGaeuiLdqKaeyizImQaeGimaadabaGaemyzau2aaWbaaSqabeaacqGHsislcqqHuoaraaaakeaacqqGVbWBcqqG0baDcqqGObaAcqqGLbqzcqqGYbGCcqqG3bWDcqqGPbqAcqqGZbWCcqqGLbqzcqGGUaGlaaaacaGL7baaaaaaaa@64AE@

The value Δ is the product of the energy difference and inverse temperature difference:

Δ : = (*β*_*j *_- *β*_*i*_)(*E*(*c*_*i*_) - *E*(*c*_*j*_)).

where βi=1Ti
 MathType@MTEF@5@5@+=feaafiart1ev1aaatCvAUfKttLearuWrP9MDH5MBPbIqV92AaeXatLxBI9gBaebbnrfifHhDYfgasaacH8akY=wiFfYdH8Gipec8Eeeu0xXdbba9frFj0=OqFfea0dXdd9vqai=hGuQ8kuc9pgc9s8qqaq=dirpe0xb9q8qiLsFr0=vr0=vr0dc8meaabaqaciaacaGaaeqabaqabeGadaaakeaaiiGacqWFYoGydaWgaaWcbaGaemyAaKgabeaakiabg2da9maalaaabaGaeGymaedabaGaemivaq1aaSbaaSqaaiabdMgaPbqabaaaaaaa@34A3@ is the inverse of the temperature of replica *i*.

Potential replica exchanges are only performed between neighbouring temperatures, since the acceptance probability of the exchange drops exponentially as the temperature difference between replicas increases.

### Our REMC algorithms

Details of our implementation of REMC search are presented in the 'Methods' section. We have experimented with three variants of the REMC algorithm for both the 2D and 3D case, which differ only in the neighbourhoods used in the subsidiary Monte Carlo local search procedure. *REMC*_*vshd *_folds protein sequences using exclusively the VSHD neighbourhood. Likewise, REMC_*pm *_is based on the pull move neighbourhood. Our third variant, REMC_*m*_, makes use of a hybrid neighbourhood that allows both, pull moves and VSHD moves to be performed; more precisely, in each local search step, the pull move neighbourhood will be used with probability *ρ *(where *ρ *is a configurable parameter of the algorithm) and otherwise, the VSHD neighbourhood will be used.

## Results

To evaluate the performance of our REMC algorithms we directly compared results against those for two state-of-the-art folding algorithms, ACO-HPPFP-3 and PERM. In the same manner in which the parameters for REMC remain fixed for all experiments, the PERM and ACO-HPPFP-3 parameters have been fixed to the values suggested by their authors. The parameter values for ACO-HPPFP-3 have been taken from Shmygelska and Hoos [9], and those for PERM were optimized by P. Grassberger and his group and pre-configued in the code kindly provided to us. For all runs of PERM, the parameter settings *β *: = 26 and *q *: = 0.2 were used [13].

In our experiments we conducted a number of runs with a given energy or CPU time cut-off on a standard set of benchmark instances for both the 2D and 3D HP protein folding problems. Furthermore, several new benchmark sets were created to evaluate the performance of REMC on long, biologically inspired sequences as well as on sequences with provably unique optimal conformations. A direct comparison between ACO-HPPFP-3 and PERM has been previously reported by Shmygelska and Hoos [9]. In this earlier work it has been shown through experiments on artificially designed as well as on known biological sequences that PERM has inherit difficulties with folding proteins where the termini interact in the formation of the hydrophobic core. Here, we performed analogous experiments to determine the performance differences between ACO-HPPFP-3, PERM and our REMC algorithms for these cases. We further tested our 2D algorithms using the pull move neighbourhood, REMC_*m *_and REMC_*pm*_, against the first algorithm based on this neighbourhood, GTabu, by means of a computational experiment analogous to that performed by Lesh *et al. *[7].

### Results for standard benchmark sequences

There are eleven benchmark sequences for the 2D HP model and ten for the 3D HP model. This benchmark set, in whole or in part, has been used extensively in the literature [8,9,11,12,45-48]. A complete listing of the 2D and 3D sequences can be found in Table 1. To evaluate performance differences between ACO-HPPFP-3, PERM and our REMC algorithms, we follow the experimental protocol established by Shmygelska and Hoos [9].

**Table 1:** Standard benchmark sequences

ID	Length	E*	Protein Sequence
2D HP			

S1-1	20	-9	(HP)_2_PH_2_PHP_2_HPH_2_P_2_HPH
S1-2	24	-9	H_2_(P_2_H)_7_H
S1-3	25	-8	P_2_HP_2_(H_2_P_4_)_3_H_2_
S1-4	36	-14	P_3_H_2_P_2_H_2_P_5_H_7_P_2_H_2_P_4_H_2_P_2_HP_2_
S1-5	48	-23	P_2_H(P_2_H_2_)_2_P_5_H_10_P_6_(H_2_P_2_)_2_HP_2_H_5_
S1-6	50	-21	H_2_(PH)_3_PH_4_PH(P_3_H)_2_P_4_H(P_3_H)_2_PH_4_(PH)_4_H
S1-7	60	-36	P_2_H_3_PH_8_P_3_H_10_PHP_3_H_12_P_4_H_6_PH_2_PHP
S1-8	64	-42	H_12_(PH)_2_(P_2_H_2_)_2_P_2_HP_2_H_2_PPH_2_P_2_HP_2_(H_2_P_2_)_2_(HP)_2_H_12_
S1-9	85	-53	H_4_P_4_H_12_P_6_(H_12_P_3_)_3_HP_2_(H_2_P_2_)_2_HPH
S1-10	100	-50	P_3_H_2_P_2_H_4_P_2_H_3_(PH_2_)_2_PH_4_P_8_H_6_P_2_H_6_P_9_HPH_2_PH_11_P_2_H_3_PH_2_PHP_2_HPH_3_P_6_H_3_
S1-11	100	-48	P_6_HPH_2_P_5_H_3_PH_5_PH_2_P_4_H_2_P_2_H_2_PH_5_PH_10_PH_2_PH_7_P_11_H_7_P_2_HPH_3_P_6_HPH_2_

3D HP			

S2-1	48	-32	HPH_2_P_2_H_4_PH_3_P_2_H_2_P_2_HPH_3_PHPH_2_P_2_H_2_P_3_HP_8_H_2_
S2-2	48	-34	H_4_PH_2_PH_5_P_2_HP_2_H_2_P_2_HP_6_HP_2_HP_3_HP_2_H_2_P_2_H_3_PH
S2-3	48	-34	PHPH_2_PH_6_P_2_HPHP_2_HPH_2_(PH)_2_P_3_H(P_2_H_2_)_2_P_2_HPHP_2_HP
S2-4	48	-33	PHPH_2_P_2_HPH_3_P_2_H_2_PH_2_P_3_H_5_P_2_HPH_2_(PH)_2_P_4_HP_2_(HP)_2_
S2-5	48	-32	P_2_HP_3_HPH_4_P_2_H_4_PH_2_PH_3_P_2_(HP)_2_HP_2_HP_6_H_2_PH_2_PH
S2-6	48	-32	H_3_P_3_H_2_PH(PH_2_)_3_PHP_7_HPHP_2_HP_3_HP_2_H_6_PH
S2-7	48	-32	PHP_4_HPH_3_PHPH_4_PH_2_PH_2_P_3_HPHP_3_H_3_(P_2_H_2_)_2_P_3_H
S2-8	48	-31	PH_2_PH_3_PH_4_P_2_H_3_P_6_HPH_2_P_2_H_2_PHP_3_H_2_(PH)_2_PH_2_P_3_
S2-9	48	-34	(PH)_2_P_4_(HP)_2_HP_2_HPH_6_P_2_H_3_PHP_2_HPH_2_P_2_HPH_3_P_4_H
S2-10	48	-33	PH_2_P_6_H_2_P_3_H_3_PHP_2_HPH_2_(P_2_H)_2_P_2_H_2_P_2_H_7_P_2_H_2_

2D and 3D standard benchmark collection which can be found in [9]. These sequences can be found originally amongst [6, 20]. As presented in [9], *H*_*i *_and *P*_*i *_indicate a string of *i *consecutive H's and P's; likewise (*s*)_*i *_indicated an *i*-fold repetition of string *s*.

Every run was performed independently with a unique random seed. In the 2D case, for sequences of length *n *≤ 50, 500 independent runs were performed; for 50 <*n *≤ 64, 100 runs; and for *n *> 64, 20 runs. In the case of 3D, 100 independent runs were performed for each sequence. Results for ACO-HPPFP-3 and PERM were taken from the study of Shmygelska and Hoos [9], which used the same experimental environment and protocol. Expected run-times for PERM are computed as texp=2⋅(1t1+1t2)−1
 MathType@MTEF@5@5@+=feaafiart1ev1aaatCvAUfKttLearuWrP9MDH5MBPbIqV92AaeXatLxBI9gBaebbnrfifHhDYfgasaacH8akY=wiFfYdH8Gipec8Eeeu0xXdbba9frFj0=OqFfea0dXdd9vqai=hGuQ8kuc9pgc9s8qqaq=dirpe0xb9q8qiLsFr0=vr0=vr0dc8meaabaqaciaacaGaaeqabaqabeGadaaakeaacqWG0baDdaWgaaWcbaGaemyzauMaemiEaGNaemiCaahabeaakiabg2da9iabikdaYiabgwSixpaabmaabaWaaSaaaeaacqaIXaqmaeaacqWG0baDdaWgaaWcbaGaeGymaedabeaaaaGccqGHRaWkdaWcaaqaaiabigdaXaqaaiabdsha0naaBaaaleaacqaIYaGmaeqaaaaaaOGaayjkaiaawMcaamaaCaaaleqabaGaeyOeI0IaeGymaedaaaaa@426F@, where *t*_1 _and *t*_2 _are the average run-times when folding from the N-terminus and C-terminus of the given protein sequence, respectively; as noted by Shmygelska and Hoos, the performance of PERM often varies substantially between folding directions [9].

Results for the 2D case are listed in Table 2. All algorithms show similar running times for the first three benchmark sequences (S1-1 to S1-3). For sequences S1-4 to S1-11, REMC_*vshd *_exhibited the worst performance; however, the other two variants of REMC, both utilizing pull moves, perform better than ACO-HPPFP-3 for all instances. PERM shows better performance than REMC_*m *_and REMC_*pm *_for sequence S1-7. On average, it also solves S1-9 faster than REMC_*pm*_. In every other case, however, REMC_*pm *_and REMC_*m *_outperform PERM, often by a significant factor. Of particular note is the fact that the variants using pull moves solve sequence S1-8 in a matter of CPU seconds compared to 78 CPU hours required on average by PERM (ACO-HPPFP-3 also outperforms PERM on this sequence with a mean running time of 1.5 CPU hours). Sequence S1-8 has a symmetric core formed by extensive interactions between the two termini; the difficulty of sequences with interacting termini for PERM has been previously demonstrated by Shmygelska and Hoos [9]. The second-hardest instance for PERM, S1-10, is solved on average 2.5 times faster by REMC_*m *_and nearly 6 times faster by REMC_*pm*_. The other benchmark sequence with 100 residues, S1-11, is solved approximately 8 times faster by both pull move variants. Overall, on the eleven benchmark instances the performance of PERM is matched or exceeded in 9 and 10 cases by REMC_*pm *_and REMC_*m*_, respectively.

**Table 2:** Results on 2D benchmark sequences

ID	E*	PERMtexp MathType@MTEF@5@5@+=feaafiart1ev1aaatCvAUfKttLearuWrP9MDH5MBPbIqV92AaeXatLxBI9gBaebbnrfifHhDYfgasaacH8akY=wiFfYdH8Gipec8Eeeu0xXdbba9frFj0=OqFfea0dXdd9vqai=hGuQ8kuc9pgc9s8qqaq=dirpe0xb9q8qiLsFr0=vr0=vr0dc8meaabaqaciaacaGaaeqabaqabeGadaaakeaacqqGqbaucqqGfbqrcqqGsbGucqqGnbqtdaWgaaWcbaGaemiDaq3aaSbaaWqaaiabdwgaLjabdIha4jabdchaWbqabaaaleqaaaaa@373A@	ACO-HPPFP-3	REMC_*vshd*_	REMC_*pm*_	REMC_*m*_
S1-1	**-9**	**-9 **(< 1 sec)	**-9 **(< 1 sec)	**-9 **(< 1 sec)	**-9 **(< 1 sec)	**-9 **(< 1 sec)
S1-2	**-9**	**-9 **(< 1 sec)	**-9 **(< 1 sec)	**-9 **(< 1 sec)	**-9 **(< 1 sec)	**-9 **(< 1 sec)
S1-3	**-8**	**-8 **(2 sec)	**-8 **(2 sec)	**-8 **(< 1 sec)	**-8 **(< 1 sec)	**-8 **(< 1 sec)
S1-4	**-14**	**-14 **(< 1 sec)	**-14 **(4 sec)	**-14 **(15 sec)	**-14 **(< 1 sec)	**-14 **(< 1 sec)
S1-5	**-23**	**-23 **(2 sec)	**-23 **(1 min)	**-23 **(91% of runs 18 min)	**-23 **(< 1 sec)	**-23 **(< 1 sec)
S1-6	**-21**	**-21 **(3 sec)	**-21 **(15 sec)	**-21 **(98% of runs 19 min)	**-21 **(< 1 sec)	**-21 **(< 1 sec)
S1-7	**-36**	**-36 **(4 sec)	**-36 **(20 min)	-34 (33% of runs 33 min)	**-36 **(10 sec)	**-36 **(13 sec)
S1-8	**-42**	**-42 **(78 hrs)	**-42 **(1.5 hrs)	-35 (11% of runs 40 min)	**-42 **(5 sec)	**-42 **(6 sec)
S1-9	**-53**	**-53 **(1 min)	**-53 **(20% of runs 1 day)	-50 (5% of runs 19 min)	**-53 **(2 min)	**-53 **(38 sec)
S1-10	**-50**	**-50 **(20 min)	-49 (12 hrs)	-46 (5% of runs 41 min)	**-50 **(3.5 min)	**-50 **(8 min)
S1-11	**-48**	**-48 **(8 min)	-47 (10 hrs)	-46 (5% of runs 97 min)	**-48 **(1 min)	**-48 **(1.2 min)

Details on runs can be found in the text. Results for PERM and ACO-HPPFP-3 are reproduced from [9]. In all instances, REMC_*vshd *_reports the worst running times followed by ACO-HPPFP-3. REMC_*m *_outperforms PERM in 10 of 11 instances and REMC_*pm *_reports better times than PERM in 9 of 11 instances. Details of the experimental protocol can be found in the text.

In the 3D case (see Table 3), the general performance trend is similar. All REMC variants report superior running times to ACO-HPPFP-3 in every case, as does PERM. Furthermore, PERM outperforms REMC_*vshd *_in each case, often by a significant factor. However, the generalization of pull moves to the 3D case has lead to a substantial increase in the performance of REMC. For only one sequence, S2-10, does PERM outperform REMC_*pm *_and REMC_*m *_(by a factor of 10). REMC using pull moves shows significantly better performance than PERM on S2-4, S2-5 and S2-9, where a five- to twenty-fold increase in performance is observed. For the other instances, REMC_*pm *_and REMC_*m *_match or outperform PERM by a small margin.

**Table 3:** Results on 3D benchmark sequences

ID	E*	PERMtexp MathType@MTEF@5@5@+=feaafiart1ev1aaatCvAUfKttLearuWrP9MDH5MBPbIqV92AaeXatLxBI9gBaebbnrfifHhDYfgasaacH8akY=wiFfYdH8Gipec8Eeeu0xXdbba9frFj0=OqFfea0dXdd9vqai=hGuQ8kuc9pgc9s8qqaq=dirpe0xb9q8qiLsFr0=vr0=vr0dc8meaabaqaciaacaGaaeqabaqabeGadaaakeaacqqGqbaucqqGfbqrcqqGsbGucqqGnbqtdaWgaaWcbaGaemiDaq3aaSbaaWqaaiabdwgaLjabdIha4jabdchaWbqabaaaleqaaaaa@373A@	ACO-HPPFP-3	REMC_*vshd*_	REMC_*pm*_	REMC_*m*_
S2-1	-32	**-32 **(0.1 min)	**-32 **(30 min)	**-32 **(0.75 min)	**-32 **(0.1 min)	**-32 **(0.1 min)
S2-2	-34	**-34 **(0.3 min)	**-34 **(420 min)	**-34 **(8.1 min)	**-34 **(0.2 min)	**-34 **(0.2 min)
S2-3	-34	**-34 **(0.1 min)	**-34 **(120 min)	**-34 **(3.3 min)	**-34 **(0.1 min)	**-34 **(0.1 min)
S2-4	-33	**-33 **(2 min)	**-33 **(300 min)	**-33 **(2.2 min)	**-33 **(0.2 min)	**-33 **(0.1 min)
S2-5	-32	**-32 **(0.5 min)	**-32 **(15 min)	**-32 **(1.2 min)	**-32 **(0.1 min)	**-32 **(0.1 min)
S2-6	-32	**-32 **(0.1 min)	**-32 **(720 min)	**-32 **(1.5 min)	**-32 **(0.1 min)	**-32 **(0.1 min)
S2-7	-32	**-32 **(0.5 min)	**-32 **(720 min)	**-32 **(3.9 min)	**-32 **(0.4 min)	**-32 **(0.3 min)
S2-8	-31	**-31 **(0.3 min)	**-31 **(120 min)	**-31 **(2.3 min)	**-31 **(0.2 min)	**-31 **(0.1 min)
S2-9	-34	**-34 **(5 min)	**-34 **(450 min)	**-34 **(14 min)	**-34 **(0.7 min)	**-34 **(0.9 min)
S2-10	-33	**-33 **(0.01 min)	**-33 **(60 min)	**-33 **(2 min)	**-33 **(0.1 min)	**-33 **(0.1 min)

Details on runs can be found in the text. Results for PERM and ACO-HPPFP-3 have been reproduced from [9]. All variants of REMC outperform ACO-HPPFP-3. REMC_*m *_and REMC_*pm *_match or outperform PERM in 9 of 10 instances. Details of the experimental protocol can be found in the text.

REMC_*pm *_and REMC_*m *_also outperform other algorithms found in the literature. Shmygelska and Hoos compared PERM and ACO-HPPFP-3 against other methods with previously published results on the standard benchmark sets [9]. For the 2D square lattice, this comparison included the genetic algorithm of Unger and Moult [11], the evolutionary Monte Carlo algorithm of Liang and Wong [8], and the multi-self-overlap ensemble algorithm of Chikenji *et al. *[47]. Furthermore, a previous application of replica exchange Monte Carlo search (parallel tempering) on the 2D square lattice [23] has failed to reach ground-state configurations for the two largest standard benchmark sequences (here referred to as S1-10 and S1-11) [47]. For the 3D cubic lattice, the hydrophobic zipper algorithm [49], the constraint-based hydrophobic core construction method [37], the core-directed chain growth algorithm [46] and the contact interactions algorithm [10] were considered. Considering these previously published results in combination with the results reported here, REMC_*pm *_and REMC_*m *_both perform better than any of the earlier methods mentioned above in terms of the energy of the conformations found or the CPU time required for reaching a given energy level (where differences in CPU speed are taken into account).

Due to their superior performance, only the REMC_*pm *_and REMC_*m *_algorithms were considered in the remainder of our study.

### Characteristic performance of REMC

Prompted by the results on sequence S1-8, we decided to further investigate to which extent REMC using pull moves can fold proteins with interacting termini in their cores substantially more effectively than PERM. To that end, we used three additional sequences that had been shown to be difficult for PERM by Shmygelska and Hoos [9]; these sequences are listed in Supplemental Table 1 [see Additional file 1]. These sequences and the corresponding mean run-times for each algorithm (determined from 100 independent runs) are reported in Table 4. For all four instances, both REMC variants outperform ACO-HPPFP-3 by factors ranging from 21 to 236. In the case of B50-5, REMC_*pm *_and REMC_*m *_easily outperform PERM (by a factor of 20) when the latter is folding from both directions or from the C-terminus; however, when folding from the N-terminus, PERM slightly outperforms REMC_*pm *_(by a factor of 1.2).

**Table 4:** Results for biological and designed sequences

ID	E*	PERMt1 MathType@MTEF@5@5@+=feaafiart1ev1aaatCvAUfKttLearuWrP9MDH5MBPbIqV92AaeXatLxBI9gBaebbnrfifHhDYfgasaacH8akY=wiFfYdH8Gipec8Eeeu0xXdbba9frFj0=OqFfea0dXdd9vqai=hGuQ8kuc9pgc9s8qqaq=dirpe0xb9q8qiLsFr0=vr0=vr0dc8meaabaqaciaacaGaaeqabaqabeGadaaakeaacqqGqbaucqqGfbqrcqqGsbGucqqGnbqtdaWgaaWcbaGaemiDaq3aaSbaaWqaaiabigdaXaqabaaaleqaaaaa@33F5@	PERMt2 MathType@MTEF@5@5@+=feaafiart1ev1aaatCvAUfKttLearuWrP9MDH5MBPbIqV92AaeXatLxBI9gBaebbnrfifHhDYfgasaacH8akY=wiFfYdH8Gipec8Eeeu0xXdbba9frFj0=OqFfea0dXdd9vqai=hGuQ8kuc9pgc9s8qqaq=dirpe0xb9q8qiLsFr0=vr0=vr0dc8meaabaqaciaacaGaaeqabaqabeGadaaakeaacqqGqbaucqqGfbqrcqqGsbGucqqGnbqtdaWgaaWcbaGaemiDaq3aaSbaaWqaaiabikdaYaqabaaaleqaaaaa@33F7@	PERMtexp MathType@MTEF@5@5@+=feaafiart1ev1aaatCvAUfKttLearuWrP9MDH5MBPbIqV92AaeXatLxBI9gBaebbnrfifHhDYfgasaacH8akY=wiFfYdH8Gipec8Eeeu0xXdbba9frFj0=OqFfea0dXdd9vqai=hGuQ8kuc9pgc9s8qqaq=dirpe0xb9q8qiLsFr0=vr0=vr0dc8meaabaqaciaacaGaaeqabaqabeGadaaakeaacqqGqbaucqqGfbqrcqqGsbGucqqGnbqtdaWgaaWcbaGaemiDaq3aaSbaaWqaaiabdwgaLjabdIha4jabdchaWbqabaaaleqaaaaa@373A@	ACO-HPPFP-3	REMC_*pm*_	REMC_*m*_
B50-5	-22	5 sec	118 sec	9 sec	820 sec	6 sec	5 sec
B50-7	-17	271 sec	299 sec	284 sec	130 sec	1 sec	2 sec
D-1	-19	3 795 sec	1 sec	2 sec	236 sec	1 sec	1 sec
D-2	-17	9 257 sec	19 356 sec	12 524 sec	951 sec	44 sec	41 sec

Results for PERM and ACO-HPPFP-3 are reproduced from [9]. In all instances, REMC finds optimal conformations relatively easily compared with the other algorithms. REMC does not demonstrate an inherit difficulty folding sequences when conformations involve hydrophobic cores confined to one end of the sequence or the case involving both ends. For every instance, 100 independent runs were conducted of 1 CPU hour each. In cases where not every run reached the same energy value after 1 hour, the expected run-time to reach the energy value shown in the table was calculated using the equation detailed by Parkes and Walser [54].

For B50-7, REMC beats all variants of PERM by more than two orders of magnitude. As B50-7 involves significant interaction between both termini, the folding direction of PERM appears to be inconsequential. This is not the case for D-1. When folding from the C-terminus, PERM has no difficulty folding the sequence within 1 CPU second, as a significant part of the C-terminus forms the hydrophobic core of this protein. This performance is matched by both REMC algorithms. However, when folding from the N-terminus, PERM requires a mean run-time of over one CPU hour. The D-2 sequence is highly symmetric in its core formation. PERM reports the worst run-times of all algorithms in this instance with a mean run-time of over 2.5 CPU hours in the best case. This is more than 200 times worse than either of the REMC algorithms. Overall, these results clearly indicate that, compared to PERM, REMC is much more effective in finding low-energy structures whose termini interact to form hydrophobic cores.

It has also been previously demonstrated that ACO-HPPFP-3 provides a larger range of relative H-H contact order values than PERM when analyzing the ensemble of folds obtained from multiple independent runs on the same sequence [9], where the ensemble contains the first optimal conformation encountered in each of the independent runs. The relative H-H contact order measures the average separation of hydrophobic-hydrophobic contacts and is formally defined as

COH−H:=1l⋅n∑i<j−1|i−j|,
 MathType@MTEF@5@5@+=feaafiart1ev1aaatCvAUfKttLearuWrP9MDH5MBPbIqV92AaeXatLxBI9gBaebbnrfifHhDYfgasaacH8akY=wiFfYdH8Gipec8Eeeu0xXdbba9frFj0=OqFfea0dXdd9vqai=hGuQ8kuc9pgc9s8qqaq=dirpe0xb9q8qiLsFr0=vr0=vr0dc8meaabaqaciaacaGaaeqabaqabeGadaaakeaacqWGdbWqcqWGpbWtdaWgaaWcbaGaemisaGKaeyOeI0IaemisaGeabeaakiabcQda6iabg2da9maalaaabaGaeGymaedabaGaemiBaWMaeyyXICTaemOBa4gaamaaqafabaWaaqWaaeaacqWGPbqAcqGHsislcqWGQbGAaiaawEa7caGLiWoaaSqaaiabdMgaPjabgYda8iabdQgaQjabgkHiTiabigdaXaqab0GaeyyeIuoakiabcYcaSaaa@49B6@

where *l *is the number H-H contacts, *n *is the number of hydrophobic residues, and *i *and *j *are hydrophobic residues in contact that are not neighbours in the chain. This measure can be employed to compare the quantity and diversity of structures returned by one or more algorithms. Since identical conformations have the same relative H-H contact order value, the number of unique structures in a set is bounded from below by the number of unique contact order values. Furthermore, a larger range of relative contact order values is indicative of a more diverse set of structures.

**Figure 4:**
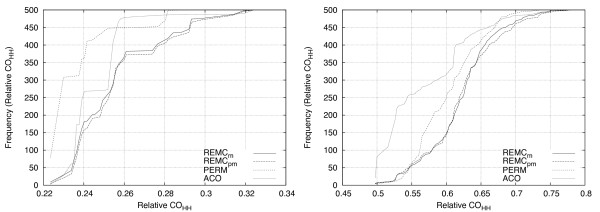
**Comparison of the distribution of H-H contact orders found by REMC, ACO-HPPFP-3 and PERM**. The frequency distribution of relative contact order values for folding S1-7 in 2D (left side) and S2-5 in 3D (right side) over 500 independent runs is shown. This measure can be employed to compare the quantity and diversity of folds returned by one or more algorithms. In both the 2D and 3D case, REMC variants have a more even distribution and find a larger number of relative contact order values than PERM or ACO-HPPFP-3. REMC and ACO-HPPFP-3 both find relative contact orders larger than PERM in 2D. In 3D, REMC finds larger relative contact order values than both PERM and ACO-HPPFP-3.

Figure 4 demonstrates the frequency distribution of relative H-H contact orders for AC0-HPPFP-3, PERM and the REMC variants using pull moves. Ground-state conformations were examined from 500 independent runs per algorithm on S1-7 for the 2D case (left side) and on S2-5 for the 3D case (right side). Runs were terminated immediately after a ground-state conformation was found. For the 2D case, ACO-HPPFP-3 and REMC find conformations with higher relative contact order than PERM does (relative *CO*_*H*-*H *_= 0.324). REMC also appears to have a flatter, more even distribution than either ACO-HPPFP-3 and PERM. Both REMC_*pm *_and REMC_*m *_find 34 unique relative contact order values, while ACO-HPPFP-3 finds 22 and PERM only 15.

In the 3D case, the REMC algorithms also find a more diverse set of ground-state structures than ACO-HPPFP-3 and PERM. REMC_*m *_and REMC_*pm *_return 82 and 83 unique values, respectively, compared to 74 found by ACO-HPPFP-3 and 69 by PERM. Furthermore, REMC finds conformation with larger relative contact order values than ACO-HPPFP-3 and PERM; the largest values are 0.789 for REMC_*m*_, 0.776 for REMC_*m*_, 0.75 for ACO-HPPFP-3 and 0.737 for PERM.

### Results for longer sequences

To evaluate how REMC's performance scales with sequence length, a new, biologically motivated test set was constructed. All sequences were taken from the Protein Data Bank and have length between 200 and 250 residues at a sequence similarity of less than 10%. Sequences were translated into HP strings based on the RASMOL hydrophobicity classification scale [50], except for non-standard amino acid symbols, such as X and Z, which were skipped (the same protocol has been previously used by Shmygelska and Hoos [9]). The resulting HP sequences are listed in Supplemental Table 2 [see Additional file 1]. As ACO-HPPFP-3 scaled poorly with sequence length on the benchmark sequences compared with PERM and REMC, it has been omitted from this evaluation. PERM was run in both directions for each instance.

**Figure 5:**
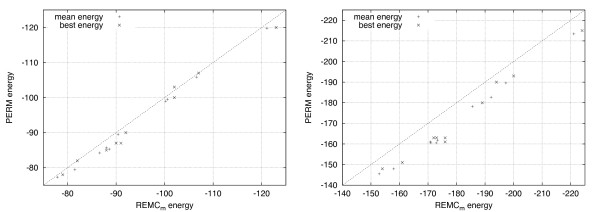
**Comparison of energies found by REMC and PERM for long biological sequences**. The best and mean energy values found over 10 independent, one hour runs for each of the long biological sequences found in Supplemental Table 2 [see Additional file 1] is shown. The mean energy values reported for each instance is the average energy found amongst the 10 independent runs; the best energy is the lowest value found amongst the 10 runs. Notice that ground-state conformations have minimal free energy and therefore lower energy values are more desirable. In the 2D case (left side), PERM finds a best energy value lower than REMC in one instance and matched the best energy value found by REMC in two other instances. In the other 7 instances, REMC finds conformations with lower energies. In all instances, the average energy found was lower for REMC than PERM. In the 3D case (right side), REMC reports lower energies than PERM overall and on average for every instance.

For each sequence, ten independent runs were conducted for each algorithm in both 2D and 3D. Runs were terminated after 60 CPU minutes on our reference machine, and the best energy was recorded. Figure 5 shows the best and mean energy values for REMC_*m *_plotted against the respective performance metrics for PERM; the best energy value corresponds to the lowest energy value found amongst all independent runs, while the mean energy value we report is the average of the best energies found in each independent run. In the 2D case (Figure 5, left side), PERM finds a better energy value for one sequence and finds the same best energy values for two others. In the remaining seven cases, REMC_*m *_finds superior conformations. For every sequence, REMC_*m *_achieves better mean energy values than PERM.

In the 3D case (Figure 5, right side), the performance difference is more pronounced. REMC finds better conformations on average and in the best case for every sequence. Considering the best conformations found among the ten independent runs for each algorithm and for each initial direction in the case of PERM, REMC_*m *_reaches significantly lower energies; the same holds with respect to average energy values. REMC_*pm *_achieves similar performance in all cases (results not shown).

### Results for sequences with unique ground-state conformations

**Figure 6:**
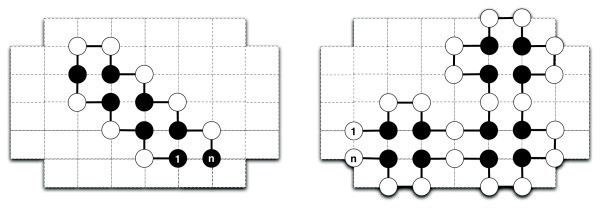
**Examples of sequence classes with unique structures**. On the left, an example of a Z-structure proposed by Aichholzer *et al. *[51] is shown. On the right, we show an example of a L1-structure proposed by Gupta *et al. *[52]. An L1-structure has one bend whereas the other L-structure we experiment with (L0) has no bends. The sequences associated with these structures have a provably unique optimal conformation [51, 52].

Further experiments were conducted on three classes of sequences with unique ground-state conformations in the 2D HP model on the square lattice. In 2003, Aichholzer *et al. *identified and proved that a class of sequences uniquely fold into structures dubbed Z-structures [51]. Later, Gupta *et al. *proposed a tile set used to design constructible structures for the inverse protein folding problem. Of these constructible structures, the authors proved that the sequences associated with linear structures with no bends (L0) and linear structures with one bend (L1) uniquely fold into the designed conformation [52]. Examples of these structures are shown in Figure 6. We constructed a new test set comprising ten sequences of increasing length for each of these classes of sequences and list them in Supplemental Table 3 [see Additional file 1]. To evaluate the performance of PERM and REMC on these test sequences, we performed 100 independent runs per sequence, each with a cut-off time of 1 CPU hour. PERM was run in both directions, and in the case where neither direction finds the (known) lowest energy, the expected run-time is reported for the best energy found.

The mean run-times for the Z-structures is reported in Table 5. REMC finds the unique conformation of each sequence relatively easily with a worst case mean run-time of 2 CPU seconds. PERM's performance, on the other hand, scales very poorly with sequence length, and the algorithm is unable to find the optimal energy for the four longest sequences.

**Table 5:** Results on stable Z-structures

ID	E*	PERMtexp MathType@MTEF@5@5@+=feaafiart1ev1aaatCvAUfKttLearuWrP9MDH5MBPbIqV92AaeXatLxBI9gBaebbnrfifHhDYfgasaacH8akY=wiFfYdH8Gipec8Eeeu0xXdbba9frFj0=OqFfea0dXdd9vqai=hGuQ8kuc9pgc9s8qqaq=dirpe0xb9q8qiLsFr0=vr0=vr0dc8meaabaqaciaacaGaaeqabaqabeGadaaakeaacqqGqbaucqqGfbqrcqqGsbGucqqGnbqtdaWgaaWcbaGaemiDaq3aaSbaaWqaaiabdwgaLjabdIha4jabdchaWbqabaaaleqaaaaa@373A@	REMC_*pm*_	REMC_*m*_
Z-4	**-3**	**-3 **(< 1 sec)	**-3 **(< 1 sec)	**-3 **(< 1 sec)
Z-8	**-7**	**-7 **(< 1 sec)	**-7 **(< 1 sec)	**-7 **(< 1 sec)
Z-12	**-11**	**-11 **(< 1 sec)	**-11 **(< 1 sec)	**-11 **(< 1 sec)
Z-16	**-15**	**-15 **(3 sec)	**-15 **(< 1 sec)	**-15 **(< 1 sec)
Z-20	**-19**	**-19 **(51 min)	**-19 **(< 1 sec)	**-19 **(< 1 sec)
Z-24	**-23**	**-23 **(49 hrs†)	**-23 **(< 1 sec)	**-23 **(< 1 sec)
Z-28	**-27**	-26	**-27 **(< 1 sec)	**-27 **(< 1 sec)
Z-32	**-31**	-29	**-31 **(< 1 sec)	**-31 **(< 1 sec)
Z-36	**-35**	-31	**-35 **(1 sec)	**-35 **(< 1 sec)
Z-40	**-39**	-34	**-39 **(2 sec)	**-39 **(1 sec)

The Z-structures proposed in [51] are easy for REMC to fold when compared with PERM. After Z-24, PERM is unable to find the unique optimal conformation in any of the 100 independent runs conducted. When only one folding direction of PERM finds the optimal conformation, we report the mean run-time of that direction, denoting this in the table with a †. When best energies found by PERMt1
 MathType@MTEF@5@5@+=feaafiart1ev1aaatCvAUfKttLearuWrP9MDH5MBPbIqV92AaeXatLxBI9gBaebbnrfifHhDYfgasaacH8akY=wiFfYdH8Gipec8Eeeu0xXdbba9frFj0=OqFfea0dXdd9vqai=hGuQ8kuc9pgc9s8qqaq=dirpe0xb9q8qiLsFr0=vr0=vr0dc8meaabaqaciaacaGaaeqabaqabeGadaaakeaacqqGqbaucqqGfbqrcqqGsbGucqqGnbqtdaWgaaWcbaGaemiDaq3aaSbaaWqaaiabigdaXaqabaaaleqaaaaa@33F5@ and PERMt2
 MathType@MTEF@5@5@+=feaafiart1ev1aaatCvAUfKttLearuWrP9MDH5MBPbIqV92AaeXatLxBI9gBaebbnrfifHhDYfgasaacH8akY=wiFfYdH8Gipec8Eeeu0xXdbba9frFj0=OqFfea0dXdd9vqai=hGuQ8kuc9pgc9s8qqaq=dirpe0xb9q8qiLsFr0=vr0=vr0dc8meaabaqaciaacaGaaeqabaqabeGadaaakeaacqqGqbaucqqGfbqrcqqGsbGucqqGnbqtdaWgaaWcbaGaemiDaq3aaSbaaWqaaiabikdaYaqabaaaleqaaaaa@33F7@ differed (and neither find the optimal solution), the best energy by either is reported and the run-time is omitted. For every instance, 100 independent runs were conducted of 1 CPU hour each. In cases where not every run reached the same energy value after 1 hour, the expected run-time to reach the energy value shown in the table was calculated using the equation detailed by Parkes and Walser [54].

The L0 structures turned out to be much more difficult to solve for REMC (see Table 6). Neither PERM nor REMC are able to find the optimal conformation for L0-9 or L0-10, although REMC_*m *_finds lower-energy conformations than PERM in both cases. PERM finds the same sub-optimal solution as REMC_*pm *_for L0-10 in significantly less time. For all other instances, both REMC variants dominate PERM by finding either lower energy conformations or by requiring less run-time for reaching the same energy values.

**Table 6:** Results on stable L0-structures

ID	E*	PERMtexp MathType@MTEF@5@5@+=feaafiart1ev1aaatCvAUfKttLearuWrP9MDH5MBPbIqV92AaeXatLxBI9gBaebbnrfifHhDYfgasaacH8akY=wiFfYdH8Gipec8Eeeu0xXdbba9frFj0=OqFfea0dXdd9vqai=hGuQ8kuc9pgc9s8qqaq=dirpe0xb9q8qiLsFr0=vr0=vr0dc8meaabaqaciaacaGaaeqabaqabeGadaaakeaacqqGqbaucqqGfbqrcqqGsbGucqqGnbqtdaWgaaWcbaGaemiDaq3aaSbaaWqaaiabdwgaLjabdIha4jabdchaWbqabaaaleqaaaaa@373A@	REMC_*pm*_	REMC_*m*_
L0-1	**-4**	**-4 **(< 1 sec)	**-4 **(< 1 sec)	**-4 **(< 1 sec)
L0-2	**-8**	**-8 **(< 1 sec)	**-8 **(< 1 sec)	**-8 **(< 1 sec)
L0-3	**-12**	**-12 **(< 1 sec)	**-12 **(< 1 sec)	**-12 **(< 1 sec)
L0-4	**-16**	**-16 **(32 sec)	**-16 **(7 sec)	**-16 **(5 sec)
L0-5	**-20**	**-20 **(3 hrs†)	**-20 **(1.1 min)	**-20 **(55 sec)
L0-6	**-24**	-23	**-24 **(16 min)	**-24 **(13 min)
L0-7	**-28**	-26 (33 sec)	**-28 **(3.2 hrs)	**-28 **(2.5 hrs)
L0-8	**-32**	-30 (3 min)	**-32 **(50 hrs)	**-32 **(16 hrs)
L0-9	**-36**	-34 (22 min)	-35 (99 hrs)	-35 (100 hrs)
L0-10	**-40**	-38 (40 min)	-38 (9.6 hrs)	-39 (100 hrs)

The L0-structures proposed in [52] are hard for both REMC and PERM to fold. After L0-5, PERM is unable to find the unique optimal conformation in any of the 100 independent runs conducted. REMC is unable to find the ground-state conformation for L0-9 and L0-10, however, REMC_*m *_finds better sub-optimal conformations than PERM in both instances. When only one folding direction of PERM finds the optimal conformation, we report the mean run-time of that direction, denoting this in the table with a †. When best energies found by PERMt1
 MathType@MTEF@5@5@+=feaafiart1ev1aaatCvAUfKttLearuWrP9MDH5MBPbIqV92AaeXatLxBI9gBaebbnrfifHhDYfgasaacH8akY=wiFfYdH8Gipec8Eeeu0xXdbba9frFj0=OqFfea0dXdd9vqai=hGuQ8kuc9pgc9s8qqaq=dirpe0xb9q8qiLsFr0=vr0=vr0dc8meaabaqaciaacaGaaeqabaqabeGadaaakeaacqqGqbaucqqGfbqrcqqGsbGucqqGnbqtdaWgaaWcbaGaemiDaq3aaSbaaWqaaiabigdaXaqabaaaleqaaaaa@33F5@ and PERMt2
 MathType@MTEF@5@5@+=feaafiart1ev1aaatCvAUfKttLearuWrP9MDH5MBPbIqV92AaeXatLxBI9gBaebbnrfifHhDYfgasaacH8akY=wiFfYdH8Gipec8Eeeu0xXdbba9frFj0=OqFfea0dXdd9vqai=hGuQ8kuc9pgc9s8qqaq=dirpe0xb9q8qiLsFr0=vr0=vr0dc8meaabaqaciaacaGaaeqabaqabeGadaaakeaacqqGqbaucqqGfbqrcqqGsbGucqqGnbqtdaWgaaWcbaGaemiDaq3aaSbaaWqaaiabikdaYaqabaaaleqaaaaa@33F7@ differed (and neither find the optimal solution), the best energy by either is reported and the run-time is omitted. For every instance, 100 independent runs were conducted of 1 CPU hour each. In cases where not every run reached the same energy value after 1 hour, the expected run-time to reach the energy value shown in the table was calculated using the equation detailed by Parkes and Walser [54].

The L1 structures are the hardest for all algorithms considered here (see Table 7). For the three longest sequences, both REMC algorithms find the same sub-optimal solutions as PERM, but PERM reaches these only for one folding direction. For the other instances, REMC consistently finds the optimal conformation significantly faster than PERM.

**Table 7:** Results on stable L1-structures

ID	E*	PERMtexp MathType@MTEF@5@5@+=feaafiart1ev1aaatCvAUfKttLearuWrP9MDH5MBPbIqV92AaeXatLxBI9gBaebbnrfifHhDYfgasaacH8akY=wiFfYdH8Gipec8Eeeu0xXdbba9frFj0=OqFfea0dXdd9vqai=hGuQ8kuc9pgc9s8qqaq=dirpe0xb9q8qiLsFr0=vr0=vr0dc8meaabaqaciaacaGaaeqabaqabeGadaaakeaacqqGqbaucqqGfbqrcqqGsbGucqqGnbqtdaWgaaWcbaGaemiDaq3aaSbaaWqaaiabdwgaLjabdIha4jabdchaWbqabaaaleqaaaaa@373A@	REMC_*pm*_	REMC_*m*_
L1-1-3	**-16**	**-16 **(120 sec)	**-16 **(6 sec)	**-16 **(6 sec)
L1-2-2	**-16**	**-16 **(57 sec)	**-16 **(3 sec)	**-16 **(2 sec)
L1-3-1	**-16**	**-16 **(28 sec)	**-16 **(3 sec)	**-16 **(3 sec)
L1-1-5	**-24**	**-24 **(100 hrs†)	**-24 **(17 min)	**-24 **(14 min)
L1-2-4	**-24**	**-24 **(49 hrs†)	**-24 **(9 min)	**-24 **(7 min)
L1-3-3	**-24**	-23	**-24 **(7 min)	**-24 **(5 min)
L1-4-2	**-24**	**-24 **(49 hrs†)	**-24 **(5 min)	**-24 **(4 min)
L1-3-7	**-40**	-38	-38 (20 hrs)	-38 (20 hrs)
L1-5-5	**-40**	-38	-38 (16 hrs)	-38 (14 hrs)
L1-8-2	**-40**	-38	-38 (16 hrs)	-38 (14 hrs)

The L1-structures proposed in [52] are the most difficult stable structures for REMC to fold. Both PERM and REMC are unable to find the optimal conformations for L1-3-7, L1-5-5, and L1-8-2 after 100, one hour runs. PERM also did not find the optimal conformation for L1-3-3. When only one folding direction of PERM finds the optimal conformation, we report the mean run-time of that direction, denoting this in the table with a †. When best energies found by PERMt1
 MathType@MTEF@5@5@+=feaafiart1ev1aaatCvAUfKttLearuWrP9MDH5MBPbIqV92AaeXatLxBI9gBaebbnrfifHhDYfgasaacH8akY=wiFfYdH8Gipec8Eeeu0xXdbba9frFj0=OqFfea0dXdd9vqai=hGuQ8kuc9pgc9s8qqaq=dirpe0xb9q8qiLsFr0=vr0=vr0dc8meaabaqaciaacaGaaeqabaqabeGadaaakeaacqqGqbaucqqGfbqrcqqGsbGucqqGnbqtdaWgaaWcbaGaemiDaq3aaSbaaWqaaiabigdaXaqabaaaleqaaaaa@33F5@ and PERMt2
 MathType@MTEF@5@5@+=feaafiart1ev1aaatCvAUfKttLearuWrP9MDH5MBPbIqV92AaeXatLxBI9gBaebbnrfifHhDYfgasaacH8akY=wiFfYdH8Gipec8Eeeu0xXdbba9frFj0=OqFfea0dXdd9vqai=hGuQ8kuc9pgc9s8qqaq=dirpe0xb9q8qiLsFr0=vr0=vr0dc8meaabaqaciaacaGaaeqabaqabeGadaaakeaacqqGqbaucqqGfbqrcqqGsbGucqqGnbqtdaWgaaWcbaGaemiDaq3aaSbaaWqaaiabikdaYaqabaaaleqaaaaa@33F7@ differed (and neither find the optimal solution), the best energy by either is reported and the run-time is omitted. For every instance, 100 independent runs were conducted of 1 CPU hour each. In cases where not every run reached the same energy value after 1 hour, the expected run-time to reach the energy value shown in the table was calculated using the equation detailed by Parkes and Walser [54].

### Comparison with GTabu

The variants of REMC utilizing pull moves significantly outperform REMC_*vshd *_for the 2D and 3D HP models. This clearly demonstrates the effectiveness of the pull move neighbourhood. To address the question to which extent the REMC search strategy contributes to the excellent performance of REMC_*pm *_and REMC_*m*_, we compared the performance of these algorithms with that of GTabu, the first algorithm for the HP model to use pull moves. In their paper describing GTabu and pull moves, Lesh *et al. *reported performance results for the standard benchmark sequences S1-8 to S1-11 [7]. To ensure the comparability of results, we used the same experimental protocol as Lesh *et al. *for evaluating our REMC algorithms on these sequences. Two hundred independent runs were performed for each sequence and the rate of successful completion (*i.e.*, fraction of runs in which the best known energy was reached) after 30 minutes and 60 minutes was reported.

Lesh *et al. *pointed out that the performance of their implementation of GTabu could be improved by a factor of 2 to 5 through relatively straightforward optimizations. Furthermore, GTabu was evaluated on different hardware (based on the 1000 MHz Alpha processor). Therefore, optimistically assuming our hardware is three times faster and GTabu performance could be improved by a factor of five, we also report run-times for GTabu if it were faster by a factor of fifteen.

**Figure 7:**
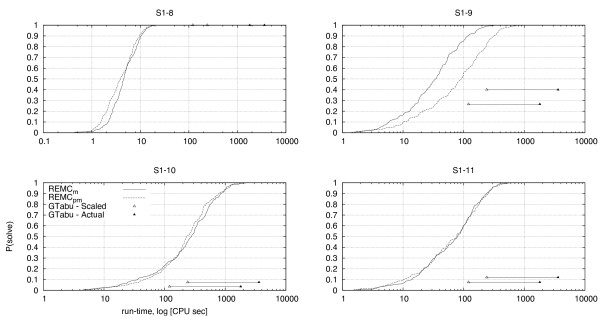
**Comparing REMC and GTabu**. The run-time distributions of REMC_*m *_and REMC_*pm *_for the four largest benchmark instances in 2D are shown; P(solve) denotes the probability of finding a ground-state conformation within a given run-time. The completion rates for GTabu after 30 minutes and 60 minutes as reported in [7] are plotted. Optimistically assuming GTabu could be improved by a factor of 15 under different experimental conditions and implementation improvements, we have also plotted the same completion rates after 2 and 4 minutes. In the case of S1-8, GTabu reports a 100% successful completion rate. In all other instances, both variants of REMC using pull moves in their local search neighbourhood outperform GTabu even under a handicapped analysis.

Figure 7 shows the run-time distributions of REMC_*pm *_and REMC_*m *_(*i.e.*, empirical distributions of run-times over the 200 independent runs) for each of the four sequences. We also indicate the completion rates achieved by GTabu after 30 CPU minutes (scaled to 2 minutes) and 60 CPU minutes (scaled to 4 minutes). Overall, even for the optimistically scaled results, it is clear that REMC significantly outperforms GTabu on three of the four instances. The remaining instance, S1-8, is the most difficult of the benchmark sequences for PERM, while being solved easily by both, GTabu and REMC.

## Discussion

In all experiments reported so far, the parameters of the REMC algorithms have remained fixed at the values listed in the 'Methods' section. These parameter sets were chosen separately for the 2D case and for the 3D case using the automatic algorithm configuration tool of Hutter et al. [53], which performs a local search in parameter space to optimize a given performance criterion (here: mean run-time). Attempts to manually configure the algorithm parameters failed to produce settings as robust as those found by the automated tool. Experiments using manually tuned parameter configurations yielded performance results that were biased in favour of either short or long sequences.

To better understand the impact of parameter settings on the performance of our REMC algorithms, we performed a series of additional experiments, in which we varied one parameter at a time, while leaving all others fixed at their default values (as specified in the 'Methods' section), *i.e.*, (*ϕ*, *τ*_*min*_, *τ*_*max*_, *χ*, *ρ*) : = (500, 160, 220, 5, 0.4) in 2D and (*ϕ*, *τ*_*min*_, *τ*_*max*_, *χ*, *ρ*) : = (500, 160, 220, 2, 0.5) in 3D, where *ϕ *is the number of local steps in a Monte Carlo search, *τ*_*min*_, and *τ*_*max*_, are the minimum and maximum temperature values respectively, *χ *is the number of replicas to simulate and *ρ *is the probability of performing a pull move.

Two test sequences were selected from the standard benchmark set for this purpose. The first sequence, S1-7, was selected for the 2D case as it does not involve significant interaction of the sequence termini in hydrophobic core formation. We did not choose a sequence with a symmetric optimal fold, such as S1-8, since in that case, REMC always appeared to find an optimal conformation fast (compared to the time required for solving other sequences of similar length), irrespectively of the parameter settings used. For the 3D case, sequence S2-7 was chosen. Neither sequence demands extensive CPU time to solve, therefore 100 independent runs were conducted for each parameter value being evaluated. In the following, we always report the mean run-time required for reaching the target energy level. Results for REMC_*vshd *_have been omitted, because they are always significantly inferior to those achieved by REMC_*m *_and REMC_*pm*_.

### Number of replicas

A particularly important parameter of any REMC algorithm is the number of replicas, *i.e.*, the number of conformations on which Monte Carlo search is concurrently performed. In the literature, the use of *χ *: = N
 MathType@MTEF@5@5@+=feaafiart1ev1aaatCvAUfKttLearuWrP9MDH5MBPbIqV92AaeXatLxBI9gBaebbnrfifHhDYfgasaacH8akY=wiFfYdH8Gipec8Eeeu0xXdbba9frFj0=OqFfea0dXdd9vqai=hGuQ8kuc9pgc9s8qqaq=dirpe0xb9q8qiLsFr0=vr0=vr0dc8meaabaqaciaacaGaaeqabaqabeGadaaakeaadaGcaaqaaiabd6eaobWcbeaaaaa@2DEC@ replicas has been suggested, where *N *is the number of degrees of freedom within the system [42].

To test the specific effect of this parameter within the context of protein folding in the HP model with our current implementation, we conducted experiments on S1-7 and S2-7 using 2, 3, 4, 5, 6, 7, 8, 9 and 10 replicas. As stated above, all other parameters remained fixed including the minimum and maximum temperature, set to 160 and 220, respectively. Formally, when evaluating the performance for replicas *χ *the temperature of replica *k*, 1 ≤ *k *≤ *χ*, was determined by the uniform linear function

T(k):=160+(k−1)⋅220−160χ−1
 MathType@MTEF@5@5@+=feaafiart1ev1aaatCvAUfKttLearuWrP9MDH5MBPbIqV92AaeXatLxBI9gBaebbnrfifHhDYfgasaacH8akY=wiFfYdH8Gipec8Eeeu0xXdbba9frFj0=OqFfea0dXdd9vqai=hGuQ8kuc9pgc9s8qqaq=dirpe0xb9q8qiLsFr0=vr0=vr0dc8meaabaqaciaacaGaaeqabaqabeGadaaakeaacqWGubavcqGGOaakcqWGRbWAcqGGPaqkcqGG6aGocqGH9aqpcqaIXaqmcqaI2aGncqaIWaamcqGHRaWkcqGGOaakcqWGRbWAcqGHsislcqaIXaqmcqGGPaqkcqGHflY1daWcaaqaaiabikdaYiabikdaYiabicdaWiabgkHiTiabigdaXiabiAda2iabicdaWaqaaGGaciab=D8aJjabgkHiTiabigdaXaaaaaa@4824@

**Figure 8:**
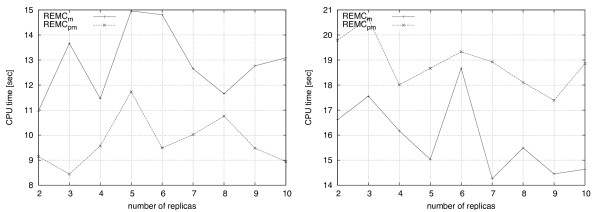
**Effect of number of replicas on run-time**. Results for mean runtimes of 100 independent runs at varying number of replicas is shown for S1-7 in 2D (left) and S2-7 in 3D (right). A general relationship is unclear, however, the runtimes observed while increasing the number of replicas scale less than the expected linear increase in most cases.

Figure 8 shows the effect of the number of replicas on mean run-time in the 2D case (left) and the 3D case (right). Interestingly, the best parameters found by the automatic algorithm configuration tool of Hutter *et al. *[53], 5 replicas for the 2D case and 2 replicas for the 3D case, seem to perform poorly for the problem instances tested here. In fact, the worst results in the 2D case for both *REMC*_*m *_and *REMC*_*pm *_occurred when using 5 replicas. However, the results shown in Figure 8 demonstrate the effect of the number of replicas on run-time only for two specific problem instances, whereas the automatic algorithm configuration tool determined parameters such that performance was optimized across a wide range of problem instances.

### Temperature distribution

**Figure 9:**
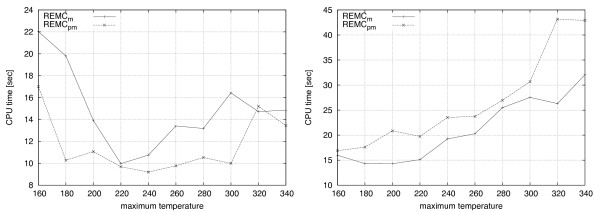
**Effect of maximum temperature on run-time**. Results for mean runtimes of 100 independent runs is shown for an increasing value of the maximum temperature. In the 2D case of folding S1-7 (left side), extremely low and extremely high temperatures yield the worst running times. The mean run-time seems to consistently scale worse as the maximum temperature is increased in the 3D case, while folding S2-7.

We now focus our attention on the effect of temperature values on run-time. The probability distributions that control the acceptance of conformations during the Monte Carlo search depend directly on the temperature settings for each replica (see Equation 1); similarly, the probability for performing replica exchanges depends on the temperature difference between neighbouring replicas (see Equation 2). Generally, a replica with a higher temperature value will accept a worsening move with a higher probability than a replica at a lower temperature. Hence, at higher temperatures, the search process is less likely to stagnate in local minima of the energy landscape. At the same time, however, lower temperatures are required for exploring promising regions effectively. Therefore, there is a trade-off between search diversification and intensification that is controlled by the temperature values used by the replicas. While our algorithms support arbitrary assignments of temperature values to each replica, in all experiments conducted in this study we have chosen simple linear temperature distributions over replicas, in which the temperature values are obtained by uniform linear interpolation between a minimum and a maximum temperature value. Furthermore, we have chosen to fix the minimum temperature to 160 in all cases; at this value, significantly worsening moves are accepted with a probability near zero while exchanges between the neighbouring temperature are still possible when reasonable values are chosen. For a thorough discussion on the use of exponential temperature distributions and the general effect of temperature distribution on performance, the reader is referred to the work of Iba [42] and Mitsutake *et al. *[24]. The results reported in Figure 9 suggest a clear relationship between maximum temperature and mean run-time in both, the 2D case (left side) and the 3D case (right side). In the 2D case, run-time is poor at both extremes of the temperature range. When temperature values are too low, the algorithm gets trapped in local minima regions for extended periods of time; likewise, higher temperatures make it difficult for the algorithm to effectively optimize promising conformations. The default parameter value of 220 seems a reasonable choice for both REMC_*m *_and REMC_*pm*_. In the case of 3D, it seems that run-time scales worse as temperature is increased.

### Number of MC steps

The parameter *φ *specifies the number of Monte Carlo steps performed by each replica between any two (proposed) replica exchanges. To determine the effect of this parameter on the run-time of our REMC algorithms, we conducted experiments using a number of values ranging from 5 to 5000 MC steps between replica exchanges.

**Figure 10:**
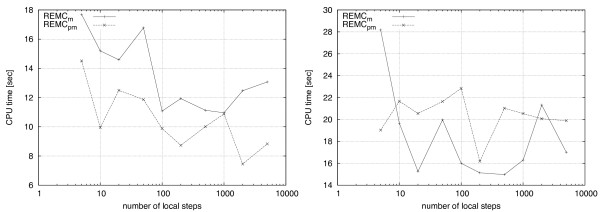
**Effect of number of local steps on run-time**. Results for mean runtimes of 100 independent runs using different numbers of local steps during Monte Carlo search, ranging from 5 to 5000, are presented. The value of local steps is plotted in log scale. Results in 2D for folding S1-7 are shown on the left and those of folding S2-7 in 3D are shown on the right. The relationship in this instance appears to be more erratic, with poorest performance often observed at the extreme values tested. The default value of 500 local steps reports good relative running times for both REMC_*m *_and REMC_*pm *_in both 2D and 3D.

Figure 10 shows the results for the 2D case (left side) and 3D case (right side). Although the relationship between the setting of *ϕ *and algorithm performance is not as clear as in the case of temperature choices, we observe that our default value of 500 MC steps is a good choice for REMC_*m *_and REMC_*pm *_on both, 2D and 3D instances.

### Probability of performing pull moves

**Figure 11:**
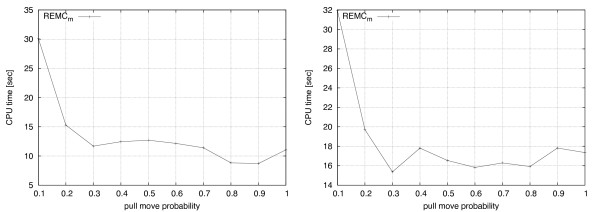
**Effect of pull move probability on run-time**. Results for mean runtimes of 100 independent runs using different probabilities of performing pull moves are reported for folding S1-7 in 2D (left) and S2-7 in 3D (right). Worst running times are observed for very low values. Otherwise, performance is generally consistent for other values.

In REMC_*m*_, a parameter *ρ *is used to specify the probability of using the pull move (rather than the VSHD) neighbourhood in any given search step. Figure 11 illustrates how the value of *ρ *affects the performance of the algorithm in the 2D case (left side) and 3D case (right side). Note that for *ρ *= 0, REMC_*m *_becomes identical to REMC_*vshd*_, and for *ρ *= 1, REMC_*m *_behaves exactly like REMC_*pm*_. As can be expected based on the results previously shown for all three algorithms, low settings of *ρ *result in substantially weaker performance than high settings; for the instances considered here, there were no significant performance differences for *ρ *≥ 0.3.

## Conclusion

In this work we have demonstrated the effectiveness of an extended ensemble algorithm, replica exchange Monte Carlo search, when applied to the protein structure prediction problem for the HP model on the two dimensional square lattice and the three dimensional cubic lattice. A direct comparison with two state-of-the-art algorithms, ACO-HPPFP-3 and PERM, on a standard set of benchmark sequences has shown that when using the pull move neighbourhood, REMC performs exceptionally well. In the 2D case, REMC ties or outperforms ACO-HPPFP-3 on every problem instance we studied. Furthermore, the performance of REMC_*m *_matches or exceeds that of PERM on ten out of the eleven benchmark instances.

In 3D, we have shown that REMC outperforms ACO-HPPFP-3 on all commonly studied benchmark instances. Moreover, REMC variants based on pull moves find ground-state conformations as fast or faster than PERM for nine of ten instances and with a mean run-time of 0.1 CPU seconds on the remaining instance (which PERM solves in 0.01 CPU seconds on average).

We have demonstrated that in the context of REMC search, using pull moves – as opposed to VSHD moves only – results in substantial performance improvements. We have also shown that by combining pull moves and VSHD moves into a hybrid search neighbourhood, better (and more robust) performance can be obtained in some cases. At the same time, the use of REMC search also contributes to the overall effectiveness of our new algorithms, as can be seen from the fact that our REMC algorithms using pull moves performs better than the GTabu algorithms, which is also based on the pull move neighbourhood. While GTabu introduced pull moves on the square lattice, (to the best of our knowledge) our study is the first to use pull moves on a 3-dimensional lattice model.

REMC proved to be very effective in folding proteins whose hydrophobic cores are formed by interacting termini, such as S1-8 from the standard benchmark set – a class of sequences that are very difficult for PERM. Similarly, we have shown that REMC finds ground-state conformations for sequences with provably unique optimal structures more effectively than PERM. We also presented evidence indicating that when applied to sequences with degenerate ground-states, REMC finds a larger and more diverse set of ground-state conformations in both 2D and 3D. Finally, we have demonstrated that REMC performs better than PERM on long biological sequences (in 2D and 3D), which suggests that REMC's performance scales quite well with sequence length. We expect, however, that for very long sequences it may be beneficial to use a chain-growth method to generate a compact conformation from which REMC search is started. Overall, we have demonstrated that REMC algorithms using the pull move neighbourhood show excellent performance on the HP model. Considering the generality of REMC and the possibility of adapting the concept of pull moves to more complex lattice structures, we see much promise in developing similar algorithms for models that can represent protein structure more accurately.

## Methods

In this section, specific details of our algorithm, experimental protocol and experimental environment are listed.

### Naming of problem instances

We have followed the naming conventions of problem instances established by Shmygelska and Hoos [9]. The instances with unique ground-state conformations were named analogously. For the long biologically-inspired sequences we retained the respective Protein Data Bank identification codes.

### Details of our REMC algorithm

**Figure 12:**
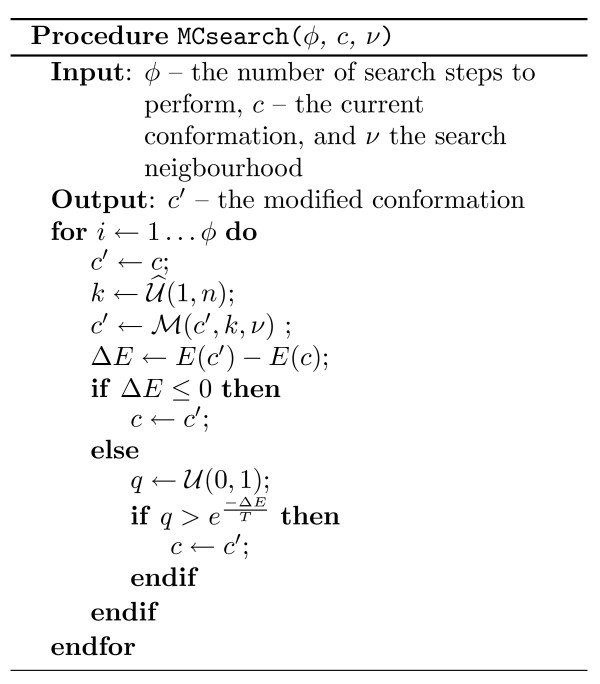
**Outline of Monte Carlo procedure**. U
 MathType@MTEF@5@5@+=feaafiart1ev1aaatCvAUfKttLearuWrP9MDH5MBPbIqV92AaeXatLxBI9gBaebbnrfifHhDYfgasaacH8akY=wiFfYdH8Gipec8Eeeu0xXdbba9frFj0=OqFfea0dXdd9vqai=hGuQ8kuc9pgc9s8qqaq=dirpe0xb9q8qiLsFr0=vr0=vr0dc8meaabaqaciaacaGaaeqabaqabeGadaaakeaat0uy0HwzTfgDPnwy1egaryqtHrhAL1wy0L2yHvdaiqaacqWFueFvaaa@3848@(*a*, *b*), and U_
 MathType@MTEF@5@5@+=feaafiart1ev1aaatCvAUfKttLearuWrP9MDH5MBPbIqV92AaeXatLxBI9gBaebbnrfifHhDYfgasaacH8akY=wiFfYdH8Gipec8Eeeu0xXdbba9frFj0=OqFfea0dXdd9vqai=hGuQ8kuc9pgc9s8qqaq=dirpe0xb9q8qiLsFr0=vr0=vr0dc8meaabaqaciaacaGaaeqabaqabeGadaaakeaadaqiaaqaamrtHrhAL1wy0L2yHvtyaeHbnfgDOvwBHrxAJfwnaGabaiab=rr8vbGaayPadaaaaa@390A@(*a*, *b*), denote a uniform random selection of a real number, and respectively an integer number, in the inclusive range *a *to *b*. ℳ
 MathType@MTEF@5@5@+=feaafiart1ev1aaatCvAUfKttLearuWrP9MDH5MBPbIqV92AaeXatLxBI9gBaebbnrfifHhDYfgasaacH8akY=wiFfYdH8Gipec8Eeeu0xXdbba9frFj0=OqFfea0dXdd9vqai=hGuQ8kuc9pgc9s8qqaq=dirpe0xb9q8qiLsFr0=vr0=vr0dc8meaabaqaciaacaGaaeqabaqabeGadaaakeaat0uy0HwzTfgDPnwy1egaryqtHrhAL1wy0L2yHvdaiqaacqWFZestaaa@378F@(*c'*, *k*, *ν*) denotes a mutation performed on conformation *c' *at residue *k*, using a move from the *ν *neighbourhood. When more than one move is feasible at position *k*, one is chosen uniformly at random.

**Figure 13:**
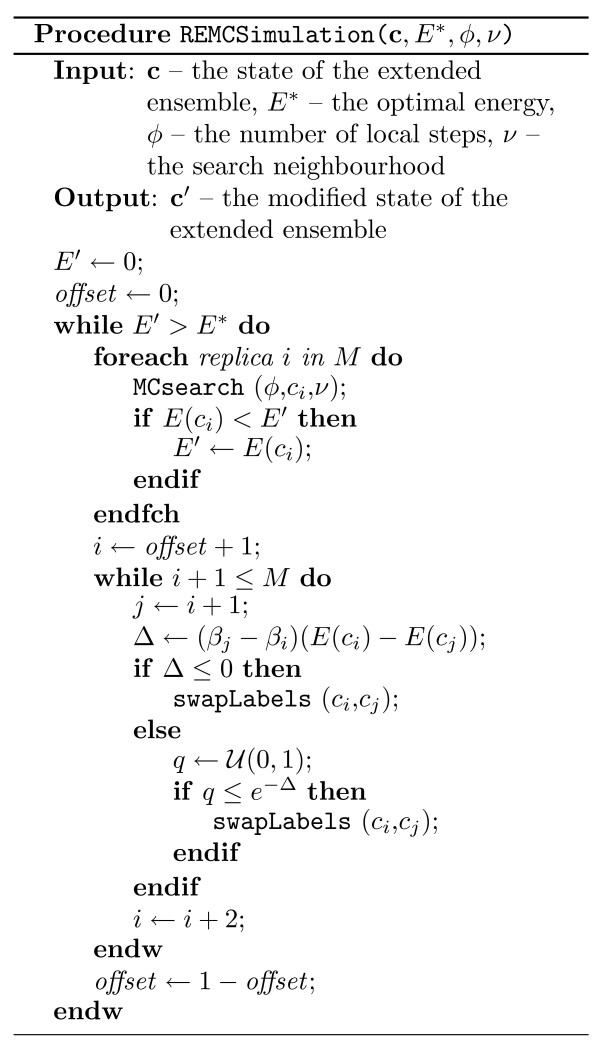
**Outline Replica exchange Monte Carlo search**. U
 MathType@MTEF@5@5@+=feaafiart1ev1aaatCvAUfKttLearuWrP9MDH5MBPbIqV92AaeXatLxBI9gBaebbnrfifHhDYfgasaacH8akY=wiFfYdH8Gipec8Eeeu0xXdbba9frFj0=OqFfea0dXdd9vqai=hGuQ8kuc9pgc9s8qqaq=dirpe0xb9q8qiLsFr0=vr0=vr0dc8meaabaqaciaacaGaaeqabaqabeGadaaakeaat0uy0HwzTfgDPnwy1egaryqtHrhAL1wy0L2yHvdaiqaacqWFueFvaaa@3848@(*a, b*) denotes a uniform random selection of a real number in the inclusive range *a *to *b*. The procedure swapLabels(*c*_*a*_, *c*_*b*_) swaps the labels (and therefore their temperature values) of replicas *a *and *b*.

In Figure 12, pseudo-code is presented illustrating the details of our Monte Carlo search procedure performed for a single replica and a predetermined number of steps. Figure 13 presents pseudo-code of our replica exchange implementation.

In order to demonstrate the effectiveness of the REMC algorithms without prior knowledge of problem instances, we have fixed parameters across all experiments including long sequences. For the 2D case, we use the parameter configuration (*ϕ*, *τ*_*min*_, *τ*_*max*_, *χ*, *ρ*) : = (500, 160, 220, 5, 0.4); in 3D, (*ϕ*, *τ*_*min*_, *τ*_*max*_, *χ*, *ρ*) : = (500, 160, 220, 2, 0.5) where *ϕ *is the number of local steps in a Monte Carlo search, *τ*_*min*_, and *τ*_*max*_, are the minimum and maximum temperature values respectively, *χ *is the number of replicas to simulate and *ρ *is the probability of performing a pull move. In the case of REMC_*vshd*_, where pull moves are not considered, we use *ρ *: = 0.0; likewise, *ρ *: = 1.0 is used in the case of REMC_*pm*_. A detailed description of these parameters and their effects on run-time can be found in the 'Discussion' section. The REMC algorithms are always run on one processor and have not been parallelized.

### Experimental protocol

In all experiments, runs were conducted independently and with unique random seeds. All runs were terminated immediately upon discovering the best known energy of some sequence, or when a pre-specified maximum run-time was exceeded in the case of fixed time runs. When less than 100% of runs were successful, *i.e.. *reached the target energy level, the expected run-time was calculated as detailed by Parkes and Walser [54] as texp:=ts+(1sr−1)⋅tf
 MathType@MTEF@5@5@+=feaafiart1ev1aaatCvAUfKttLearuWrP9MDH5MBPbIqV92AaeXatLxBI9gBaebbnrfifHhDYfgasaacH8akY=wiFfYdH8Gipec8Eeeu0xXdbba9frFj0=OqFfea0dXdd9vqai=hGuQ8kuc9pgc9s8qqaq=dirpe0xb9q8qiLsFr0=vr0=vr0dc8meaabaqaciaacaGaaeqabaqabeGadaaakeaacqWG0baDdaWgaaWcbaGaemyzauMaemiEaGNaemiCaahabeaakiabcQda6iabg2da9iabdsha0naaBaaaleaacqWGZbWCaeqaaOGaey4kaSYaaeWaaeaadaWcaaqaaiabigdaXaqaaiabdohaZjabdkhaYbaacqGHsislcqaIXaqmaiaawIcacaGLPaaacqGHflY1cqWG0baDdaWgaaWcbaGaemOzaygabeaaaaa@4500@, where *t*_*s *_and *t*_*f *_are the mean run-time of successful and unsuccessful runs, respectively, and *sr *is the ratio of successful to unsuccessful runs. All timing of runs was performed measuring CPU time and started with the first search step.

### Experimental environment

All experiments were performed on PCs with 2.4Ghz Pentium IV processors with 256KB cache and 1GB of RAM, running SUSE Linux version 9.2, unless explicitly stated otherwise.

### Implementation

All versions of our REMC protein folding algorithms were coded in C++ and compiled using g++ (GCC version 3.3.3). The source code is freely available under the GNU General Public License (GPL) and can be downloaded from our project website [55].

## Authors' contributions

HH and AS initially proposed to investigate REMC in combination with the pull move neighbourhood for the HP folding problem. AS provided code which partially formed the basis of the initial REMC implementation. CT implemented REMC and conducted all experiments. CT and HH collaborated on improving REMC, incorporating pull moves and generalizing them to the 3D cubic lattice; they also designed most of the computational experiments. All authors were involved in interpreting experimental results and in writing this manuscript.

## Supplementary Material

Additional file 1 Supplemental material. This file contains tables listing the biologically motivated benchmark sets and the problems instances with a provably unique ground-state conformation. Additionally, results of simulations are reported for the rate of energy evaluations (per CPU second) achieved by our implementation. Click here for file

## References

[B1] Berger B, Leighton T (1998). Protein folding in the hydrophobic-hydrophilic (HP) is NP-complete. Proceedings of the second annual international conference on Computational molecular biology.

[B2] Crescenzi P, Goldman D, Papadimitriou C, Piccolboni A, Yannakakis M (1998). On the complexity of protein folding. Proceedings of the second annual international conference on Computational molecular biology.

[B3] Hart W, Istrail S (1997). Robust proofs of NP-hardness for protein folding: general lattices and energy potentials. Journal of Computational Biology.

[B4] Grassberger P (1997). Pruned-enriched Rosenbluth method: Simulations of *θ *polymers of chain length up to 1 000 000. Physical review E, Statistical physics, plasmas, fluids, and related interdisciplinary topics.

[B5] Gront D, Kolinski A, Skolnick J (2001). A new combination of replica exchange Monte Carlo and histogram analysis for protein folding and thermodynamics. The Journal of Chemical Physics.

[B6] Konig R, Dandekar T (1999). Improving genetic algorithms for protein folding simulations by systematic crossover. Biosystems.

[B7] Lesh N, Mitzenmacher M, Whitesides S (2003). A complete and effective move set for simplified protein folding. RECOMB '03: Proceedings of the seventh annual international conference on Research in computational molecular biology.

[B8] Liang F, Wong WH (2001). Evolutionary Monte Carlo for protein folding simulations. The Journal of Chemical Physics.

[B9] Shmygelska A, Hoos H (2005). An ant colony optimisation algorithm for the 2D and 3D hydrophobic polar protein folding problem. BMC Bioinformatics.

[B10] Toma L, Toma S (1996). Contact interactions method: A new algorithm for protein folding simulations. Protein Science.

[B11] Unger R, Moult J (1993). Genetic Algorithms for Protein Folding Simulations. Journal of Molecular Biology.

[B12] Unger R, Moult J (1993). Genetic Algorithm for 3D Protein Folding Simulations. Proceedings of the 5th International Conference on Genetic Algorithms.

[B13] Hsu HP, Mehra V, Nadler W, Grassberger P (2003). Growth-based optimization algorithm for lattice heteropolymers. Physical review E, Statistical, nonlinear, and soft matter physics.

[B14] Bastolla U, Frauenkron H, Grassberger P (2000). Phase Diagram of Random Heteropolymers: Replica Approach and Application of a New Monte Carlo Algorithm. Journal of Molecular Liquids.

[B15] Dorigo M, Gambardella LM (1997). Ant Colony System: A Cooperative Learning Approach to the Traveling Salesman Problem. IEEE Transactions on Evolutionary Computation.

[B16] Klau GW, Lesh N, Marks J, Mitzenmacher M (2002). Human-guided tabu search. Eighteenth national conference on Artificial intelligence.

[B17] Gront D, Kolinski A, Skolnick J (2000). Comparison of three Monte Carlo conformational search strategies for a proteinlike homopolymer model: Folding thermodynamics and identification of low-energy structures. The Journal of Chemical Physics.

[B18] Hilhorst HJ, Deutch JM (1975). Analysis of Monte Carlo results on the kinetics of lattice polymer chains with excluded volume. The Journal of Chemical Physics.

[B19] Kremer K, Binder K (1988). Monte Carlo simulation of lattice models for macromolecules. Computer Physics Reports.

[B20] Ramakrishnan R, Ramachandran B, Pekny JF (1997). A dynamic Monte Carlo algorithm for exploration of dense conformational spaces in heteropolymers. The Journal of Chemical Physics.

[B21] Hansmann UHE (1997). Parallel tempering algorithm for conformational studies of biological molecules. Chemical Physics Letters.

[B22] Irbäck A, Sandelin E (1999). Monte Carlo study of the phase structure of compact polymer chains. The Journal of Chemical Physics.

[B23] Irbäck A, Grassberger P, Barkema G, Nadler W (1998). Dynamic Parameter Algorithms for Protein Folding. Monte Carlo Approach to Biopolymers and Protein Folding.

[B24] Mitsutake A, Sugita Y, Okamoto Y (2001). Generalized-ensemble algorithms for molecular simulations of biopolymers. Peptide Science.

[B25] Geyer C, Keramidas E (1991). Markov chain Monte Carlo maximum likelihood. Computing Science and Statistics: Proceedings of the 23rd Symposium on the Interface.

[B26] Hukushima K, Nemoto K (1996). Exchange Monte Carlo Method and Application to Spin Glass Simulations. Journal of the Physical Society of Japan.

[B27] Iba Y (1993). Review of Extended Ensemble Algorithms. Proceedings of the Institute of Statistical Mathematics.

[B28] Kimura K, Taki K, Vichnevetsky R, Miller JJH (1991). Time-homogeneous parallel annealing algorithm. Proceedings of the 13th IMACS World Congress on Computation and Applied Mathematics (IMACS'91).

[B29] Hukushima K, Takayama H, Yoshino H (1998). Exchange Monte Carlo Dynamics in the SK Model. Journal of the Physical Society of Japan.

[B30] Lin CY, Hu CK, Hansmann UH (2003). Parallel tempering simulations of HP-36. Proteins.

[B31] Sugita Y, Kitao A, Okamoto Y (2000). Multidimensional replica-exchange method for free-energy calculations. The Journal of Chemical Physics.

[B32] Sugita Y, Okamoto Y (1999). Replica-exchange molecular dynamics method for protein folding. Chemical Physics Letters.

[B33] Sugita Y, Okamoto Y (2001). Free-Energy Calculations in Protein Folding by Generalized-Ensemble Algorithms. Lecture Notes in Computational Science and Engineering.

[B34] Sugita Y, Okamoto Y (2000). Replica-exchange multicanonical algorithm and multicanonical replica-exchange method for simulating systems with rough energy landscape. Chemical Physical Letters.

[B35] Dill KA (1985). Theory for the folding and stability of globular proteins. Biochemistry.

[B36] Lau KF, Dill KAD (1989). A lattice statistical mechanics model of the conformational and sequence spaces of proteins. Macromolecules.

[B37] Yue K, Dill K (1995). Forces of Tertiary Structural Organization in Globular Proteins. Proceedings of the National Academy of Sciences of the United States of America.

[B38] Kolinski A, Skolnick J (2004). Reduced models of proteins and their applications. Polymer.

[B39] Dill KA, Bromberg S (2003). Molecular Driving Forces.

[B40] Verdier PH, Stockmayer WH (1962). Monte Carlo Calculations on the Dynamics of Polymers in Dilute Solution. The Journal of Chemical Physics.

[B41] Gurler MT, Crabb CC, Dahlin DM, Kovac J (1983). Effect of bead movement rules on the relaxation of cubic lattice models of polymer chains. Macromolecules.

[B42] Iba Y (2001). Extended Ensemble Monte Carlo. International Journal of Modern Physics C.

[B43] Marinari E, Parisi G (1992). Simulated tempering: a new Monte Carlo scheme. Europhysics Letters.

[B44] Swendsen R, Wang J (1986). Replica Monte Carlo simulation of spin glasses. Physical Review Letters.

[B45] Bastolla U, Frauenkron H, Gerstner E, Grassberger P, Nadler W (1998). Testing a new Monte Carlo algorithm for protein folding. Proteins.

[B46] Beutler TC, Dill KA (1996). A fast conformational search strategy for finding low energy structures of model proteins. Protein Science.

[B47] Chikenji G, Kikuchi M, Iba Y (1999). Multi-Self-Overlap Ensemble for Protein Folding: Ground State Search and Thermodynamics. Physical Review Letters.

[B48] Krasnogor N, Hart WE, Smith J, Pelta DA, Banzhaf W, Daida J, Eiben AE, Garzon MH, Honavar V, Jakiela M, Smith RE (1999). Protein Structure Prediction With Evolutionary Algorithms. Proceedings of the Genetic and Evolutionary Computation Conference.

[B49] Dill K, Fiebig K, Chan H (1993). Cooperativity in Protein-Folding Kinetics. Proceedings of the National Academy of Sciences of the United States of America.

[B50] Sayle R, Milner-White EJ (1995). RASMOL – Biomolecular Graphics for All. Trends in biochemical sciences.

[B51] Aichholzer O, Bremner D, Demaine ED, Meijer H, Sacristan V, Soss M (2003). Long proteins with unique optimal foldings in the H-P model. Computational Geometry.

[B52] Gupta A, Manuch J, Stacho L (2005). Structure-Approximating Inverse Protein Folding Problem in the 2D HP Model. Journal of Computational Biology.

[B53] Hutter F, Hoos HH, Stützle T (2007). Automatic Algorithm Configuration based on Local Search. Proceedings of the Twenty-Second Conference on Artifical Intelligence (AAAI '07).

[B54] Parkes A, Walser J (1996). Tuning Local Search for Satisfiability Testing. Proceedings of the Application of Artifical Intelligence Conference.

[B55] A replica exchange Monte Carlo algorithm for protein folding in the HP model: Project website. http://www.cs.ubc.ca/labs/beta/Projects/REMC-HPPFP.

