# Supplementary material for: A replica exchange Monte Carlo algorithm for protein folding in the HP model
Source: BMC Bioinformatics. 2007 Sep 17;8:342. doi: 10.1186/1471-2105-8-342 (PMC2071922; doi:10.1186/1471-2105-8-342)
Supplement: Additional file 1 — Supplemental material. This file contains tables listing the biologically motivated benchmark sets and the problems instances with a provably unique ground-state conformation. Additionally, results of simulations are reported for the rate of energy evaluations (per CPU second) achieved by our implementation. [file 1471-2105-8-342-S1.pdf]

# Supplemental Material

## Supplemental Table 1 - Biological and designed sequences

Biological and designed sequences originally found [1]. This set was originally used to test the hypothesis that PERM has difficulty folding sequences in which both termini interact to form the structural hydrophobic core. The sequences are re-evaluated in this study to determine if REMC has any inherit bias for folding sequences with this property.

| ID | Len | $E^*$ | Protein Sequence |
|---|---|---|---|
| B50-5 | 53 | -22 | $PHP_2H_2(HPP)_2PH_3PHP_2H_3P_3H_3PH_2(PPH)_3PH_2P_3H_2(PH)_2$ |
| B50-7 | 45 | -17 | $P_2HPH_2PH_3PH_2P_2H_4(PH)_2HP_5H_4P_7H_2P_3$ |
| D-1 | 50 | -19 | $PHP_8H_2P_4H_2P_2H_4P_4H_2P_6H_2P_2HP_2H_6P$ |
| D-2 | 60 | -17 | $PHP_4HPH_4P_6H_4P_3(HP_6)_2P_4H_3P_4H_2P_6HP$ |

## Supplemental Table 2 - Long biological sequences

A set of ten sequences exhibiting less than 10% sequence similarity was selected from the Protein Data Bank with each sequence having length between 200 and 250 amino acids. Sequence IDs correspond to the Protein Databank ID. The sequences were translated into HP strings following the protocol originally used in [1].

| ID | Len | Protein Sequence |
|---|---|---|
| 1B0F | 218 | HHHHPPHPHPHHHHHHPHPHHHHPHHHHPHHHHPHHHHPHH<br>PHHHPHPHPHHPHHHHHPPHPPPPHPPPHHHHPPHHPPHP<br>PHHPHHPPHHHHPHPHPHPHPHPHPHHPHHHPHPPHHHPHH<br>PHHHHHHHHHHPPPHHHPHHPPHPHPHHPPHHPPPPHHPH<br>HPHPPHHHHHHPPHPHHHHPHHHPHHHPHHPHHHHPHHPH<br>PHHHHHHPHHPHHPPHHP |
| 1BQS | 209 | HPHHPHPHHPHHHHHHHHHHPPPHPHPHHHHPPHHPHPHPH<br>HPPPHHHHPPPPHPPHHPHPPHPHPHHHPPHHHHHPHHHPP<br>HPPPHPHHHPHHHPPHPHPHHHHHHHHPHPHHHPHPPHPHH<br>PHPHHPHPHHHHHPPHPHHPHHHHPHPPPPPPHPHPPPHH<br>HPHPPPHPHHHHHPHHHHHHPHPHPHPHHHHPHPPPPHHH<br>HHPPHPPHP |
| 1CWR | 211 | HHPPPPHHPPPHPPHHPPPPPPHHHPPPPHHHHPPHPHPP<br>PPHPPHPHPPHHHHHPHPHPPHHPPHHHHPHPPHPPPPHH<br>PHPPHHPHHHPPPHPPHHHPPHHHHHHPHPPPHHHHHPHPH<br>PPPPHHPHHPHHPPPPPHHPPPHPHPPHPHPHHPHHPPPH<br>HHPPHPHHPHPPPPHHPPPHHPPPHPPHPHPPPPHHHPPP<br>PPPHHPPHPPP |
| 1NQC | 217 | HHPPHPHPPPHHHPPHPPPHPHHHHHHHPHHHHHHPHPHPH<br>PPHPHHPHPHPPHHPHPPPPPHPPHHPHHHHHPPHHPPHHP<br>PPHHPPPHPPHPPHHPPPHPPPPPPPHHPHPPPPPHHPHP<br>PPHHPPHHHPPHHHPHHHPHPPHPHHHPPPHHPPPHPHPP<br>PHPPHHHHHHPHPHPHPPPHHHPPPHHPPHHPPHPHPHHP<br>PPHPPHHHHPHHPPHPH |
| 1RTG | 210 | HPHHPHHPHPHHHHPHPHHPPPHHHPHHHPHPHPHHHHPP<br>PHHHPPHPHPPPHHHHHHHHHPHHHPHHPPHPHHPPHHPPP<br>PHHHHHHPPPHHPPHPPHPPHPHPHHPPHHHHHPHPPHPH<br>HHPHPPPPPPPHHHHPPHHPPPPHPPPHPHHHHPHHHPHH<br>PHHHPPHPHHHPHPHHHPPPHHPHHPPHPHPPPPHPPHPH<br>HPHPPPHHHH |

| ID | Len | Protein Sequence |
|---|---|---|
| 2GPQ | 217 | HHPHPHPPPHPHPHHPPPPPPPPPPPPPHHPHPPPHPPHHP<br>PPHHHHHHPPPPPPPPHPHPHPHHPPHPPHPPHHHHPPPHP<br>HPPPHHHHHPPPHHPPHHPHHHPPPPPPPPHHPHHHPHPPP<br>PPPPPHPPHHHPPHHHHHHPPHPPPPPPPHHHHHHPHPHPH<br>PPHHHHPPPHPPPPHHPPHHPHPPPPHHHHHPHHHHPPPP<br>HPPHPPPHPPPPPPPHHH |
| 1BEC | 238 | HHPPPHPPPHHHPHHPHPHPHPPPPPPPPPHPHPPPPPPHPH<br>HPHHPPPPPHHHPPPPPHPHHPHPPHPPHPPPPPHPHHHPHHP<br>HPPPPHPHHHPHHHPHPPHPPHHHHHPPHPHHPPHPPHPH<br>HPHPHHPHPPHPHHPPPPHPHHHHHPHHHHPPHPHPHHHP<br>HPPHPPHHPPPHPHPPPPPPPPPHHPPPHPHPHPHHPPHPP<br>PHPHPHPHPHHPPPPPHHPHPHPHHPPPHPHPHHHPHP |
| 1BPB | 248 | HPPPPPPPPHPHHPPHPHHHHPHHPPHHPPHHPPHPPHPP<br>PPPPHPPPPPHHHPPHPPHPPPHHPPPHHPHPPHHHPPHP<br>PHPHPPHHPHHHPHPPHHPPPHPHPHHHPPHPHPPPPPPP<br>HPHHPPHHPPHPPHPHHPPPHPPHPPPHHHHHPHHPPPPP<br>PPPHPPPHPHPHHHPPPPPHHHHPHPHPPHHPPPHPHPHH<br>PPHHPHPPPPHPHHHHPHHHHPHHHHPPPPPHHPPHPHPP<br>PPHPPPPP |
| 1DUA | 242 | PHPHPPHPPPPPPHPPPPHHPHHPHHPPHHPPHPPPPPPP PH-<br>PPPHHPHHHPHPPPPHPHHHHHPPPHHPPPPHHPHHPHP HPPH-<br>PHPHPHPPPPPHPPPPHPHPPPHPPHHPPHHHHHHP HHHHPH-<br>PHPPPHHPHHPPHPHHPHHPPPPHPPHPPHPHHH PHHPPPHP-<br>PHPPPPHPHPPHPPHHPPPHHPHHHPPHPHHH PPPHPHHHH-<br>PPPPHPPHPPPPPPHPPHHHHHHPPHPPHHPPP PP |
| 1FBN | 230 | HPPHPHPPHHPPHPPHPHHPHHPPHHPPPHHPHPPHPPPP<br>HHPHHPPPPPPHHPHPPPPPHHHHHHPHHPHHHHPPPPPPHHP<br>HHHPHHPPHPPHHPHHPPHHHPHHPPHHPHHPPHHPHHHP<br>PPPHHHHHHPHPPHPPPHPHHPPHPHHPPPHHPHPPHPHH<br>HPPHPHHHPPHHPHHHHHPHPPHPHPPPHPPHHPPPPPHH<br>PHHHHPHHPPHPHPHHPPPPHHHHHHHHPHP |

## Supplemental Table 3 - Stable structure sequences

The sequences listed below have provably unique optimal conformations in the HP model on the 2D square lattice. Z-structures are described in [2] and L0 and L1 structures are presented in [3].

| ID | Length | $E^*$ | Protein Sequence |
|---|---|---|---|
| | | | Z-Sequences |
| Z-4 | 8 | -3 | $(HP)_2(PH)_2$ |
| Z-8 | 16 | -7 | $(HP)_4(PH)_4$ |
| Z-12 | 24 | -11 | $(HP)_6(PH)_6$ |
| Z-16 | 32 | -15 | $(HP)_8(PH)_8$ |
| Z-20 | 40 | -19 | $(HP)_{10}(PH)_{10}$ |
| Z-24 | 48 | -23 | $(HP)_{12}(PH)_{12}$ |
| Z-28 | 56 | -27 | $(HP)_{14}(PH)_{14}$ |
| Z-32 | 64 | -31 | $(HP)_{16}(PH)_{16}$ |
| Z-36 | 72 | -35 | $(HP)_{18}(PH)_{18}$ |
| Z-40 | 80 | -39 | $(HP)_{20}(PH)_{20}$ |
| | | | L0-Sequences |
| L0-1 | 12 | -4 | $P(HPPHP)_1(PHPPH)_1P$ |
| L0-2 | 22 | -8 | $P(HPPHP)_2(PHPPH)_2P$ |
| L0-3 | 32 | -12 | $P(HPPHP)_3(PHPPH)_3P$ |
| L0-4 | 42 | -16 | $P(HPPHP)_4(PHPPH)_4P$ |
| L0-5 | 52 | -20 | $P(HPPHP)_5(PHPPH)_5P$ |
| L0-6 | 62 | -24 | $P(HPPHP)_6(PHPPH)_6P$ |
| L0-7 | 72 | -28 | $P(HPPHP)_7(PHPPH)_7P$ |
| L0-8 | 82 | -32 | $P(HPPHP)_8(PHPPH)_8P$ |
| L0-9 | 92 | -36 | $P(HPPHP)_9(PHPPH)_9P$ |
| L0-10 | 102 | -40 | $P(HPPHP)_{10}(PHPPH)_{10}P$ |
| | | | L1-Sequences |
| L1-1-3 | 42 | -16 | $P(HPPHP)_1PHP(HPPHP)_3(PHPPH)_3PHP$ |
| L1-2-2 | 42 | -16 | $P(HPPHP)_2PHP(HPPHP)_2(PHPPH)_2PH(PHPPH)_1P$ |
| L1-3-1 | 42 | -16 | $P(HPPHP)_3PHP(HPPHP)_1(PHPPH)_1PH(PHPPH)_2P$ |
| L1-1-5 | 42 | -24 | $P(HPPHP)_1PHP(HPPHP)_5(PHPPH)_5PHP$ |
| L1-2-4 | 62 | -24 | $P(HPPHP)_2PHP(HPPHP)_4(PHPPH)_4PH(PHPPH)_1P$ |
| L1-3-3 | 62 | -24 | $P(HPPHP)_3PHP(HPPHP)_3(PHPPH)_3PH(PHPPH)_2P$ |
| L1-4-2 | 62 | -24 | $P(HPPHP)_4PHP(HPPHP)_2(PHPPH)_2PH(PHPPH)_3P$ |
| L1-3-7 | 102 | -40 | $P(HPPHP)_3PHP(HPPHP)_7(PHPPH)_7PH(PHPPH)_2P$ |
| L1-5-5 | 102 | -40 | $P(HPPHP)_5PHP(HPPHP)_5(PHPPH)_5PH(PHPPH)_4P$ |
| L1-8-2 | 102 | -40 | $P(HPPHP)_8PHP(HPPHP)_2(PHPPH)_2PH(PHPPH)_7P$ |

## Supplemental Table 4 - Energy evaluations per CPU second

For each standard benchmark sequence, 20 independent runs lasting exactly 5 minutes were conducted to determine the rate of energy evaluations per CPU second for both REMC$_{vshd}$ and REMC$_{pm}$. Each run lasted 5 minutes and was not terminated if the ground state structure was found before that time. The parameters were fixed to the default settings for each run as specified in the methods section of the main article. This information is presented to help facilitate performance comparisons of our work to other algorithms based on the number of energy evaluations. The mean number of energy evaluations per CPU second over the 20 trials is reported for each standard benchmark sequence along with the standard deviation in brackets for both variants of the algorithm.

| ID | Length | Mean energy evaluations per CPU second (standard deviation) | |
|---|---|---|---|
| | | REMC$_{vshd}$ | REMC$_{pm}$ |
| S1-1 | 20 | 592395 (72216) | 363645 (22007) |
| S1-2 | 24 | 574165 (66820) | 372645 (32543) |
| S1-3 | 25 | 614195 (45212) | 368685 (23479) |
| S1-4 | 36 | 601265 (59782) | 377800 (33550) |
| S1-5 | 48 | 589230 (59699) | 390860 (39808) |
| S1-6 | 50 | 582160 (42229) | 365975 (23975) |
| S1-7 | 60 | 623060 (63967) | 369960 (35678) |
| S1-8 | 64 | 588540 (48504) | 454000 (33743) |
| S1-9 | 85 | 594510 (66470) | 384675 (42416) |
| S1-10 | 100 | 567645 (69344) | 375480 (39449) |
| S1-11 | 100 | 580625 (40855) | 379535 (41179) |
| S2-1 | 48 | 1835850 (167110) | 607765 (41233) |
| S2-2 | 48 | 1884300 (124368) | 613120 (55769) |
| S2-3 | 48 | 1852150 (118972) | 618670 (57270) |
| S2-4 | 48 | 1832900 (159192) | 598220 (55186) |
| S2-5 | 48 | 1837500 (162002) | 598840 (38564) |
| S2-6 | 48 | 1838750 (165123) | 581800 (54224) |
| S2-7 | 48 | 1862550 (113022) | 584130 (53374) |
| S2-8 | 48 | 1856750 (161765) | 587165 (39950) |
| S2-9 | 48 | 1840150 (163281) | 596115 (54496) |
| S2-10 | 48 | 1841550 (164147) | 603355 (54944) |

# References


[1] Shmygelska A, Hoos H: **An ant colony optimisation algorithm for the 2D and 3D hydrophobic polar protein folding problem**. *BMC Bioinformatics* 2005, **6**:30.

[2] Aichholzer O, Bremner D, Demaine ED, Meijer H, Sacristan V, Soss M: **Long proteins with unique optimal foldings in the H-P model**. *Computational Geometry* 2003, **25**(1-2):139–159.

[3] Gupta A, Manuch J, Stacho L: **Structure-Approximating Inverse Protein Folding Problem in the 2D HP Model**. *Journal of Computational Biology* 2005, **12**(10):1328–1345.
